# Lactancia materna entre rejas: experiencias de las madres encarceladas en el sistema penitenciario español

**DOI:** 10.18294/sc.2024.4665

**Published:** 2024-02-29

**Authors:** Pilar Jubany-Roig, Ester Massó Guijarro

**Affiliations:** 1 Psicóloga. Investigadora predoctoral, Programa de Doctorado en Filosofía, Universidad de Granada, España. proigjubany@correo.ugr.es Universidad de Granada Programa de Doctorado en Filosofía Universidad de Granada Spain proigjubany@correo.ugr.es; 2 Doctora en Filosofía y Antropología. Profesora titular de Filosofía Moral, Universidad de Granada, España. ester@ugr.es Universidad de Granada Universidad de Granada Spain ester@ugr.es

**Keywords:** Cárceles, Lactancia Materna, Violencia Obstétrica, Derechos Humanos, Feminismo, Interseccionalidad, Salud Mental, España

## Abstract

Esta investigación tiene como objetivo analizar la experiencia con respecto a la lactancia materna de las madres encarceladas en las prisiones del sistema penitenciario español, así como estudiar si han percibido prácticas que aludan a la violencia obstetricia durante la gestación, el parto y el puerperio. Se realizó un estudio exploratorio-descriptivo con abordaje cualitativo y método etnográfico crítico. Entre diciembre de 2021 y abril de 2022, se efectuó el trabajo de campo con observación participante y entrevistas semiestructuradas a 30 de las mujeres mayores de edad procedentes de África, Europa, Europa del Este y Latinoamérica, que se encontraban cumpliendo condena junto a sus criaturas en las Unidades de Madres de las ciudades españolas de Alicante, Barcelona, Madrid y Sevilla. Las principales conclusiones señalan la necesidad de aplicar políticas penitenciarias con perspectiva de género y feminista, que consigan erradicar las graves desigualdades y discriminaciones que sufren las mujeres encarceladas y que sirvan para proteger los derechos básicos de madres y criaturas.

## INTRODUCCIÓN

Las cárceles femeninas, así como la situación de las mujeres encarceladas en el contexto español, no han sido objeto de estudio en profundidad hasta finales de la década de 1980^1^, tratándose hasta entonces de un ámbito tradicionalmente ignorado, con una flagrante laguna bibliográfica.

La cuestión de la lactancia materna, por su parte, sí ha ameritado interés desde ámbitos bien distintos, habiéndose generado marcos de conocimiento importantes al respecto vinculados a la maternidad y la crianza^2^. No obstante, es limitada la bibliografía que vincule ambas cuestiones, es decir, que haga referencia a la lactancia materna en el ámbito penitenciario español, con relación a las investigaciones realizadas en Brasil^3^^,^^4^^,^^5^^,^^6^^,^^7^^,^^8^^,^^9^^,^^10^ o en EEUU^11^^,^^12^^,^^13^^,^^14^, por ejemplo.

Por lo tanto, se ha detectado un hueco fundamental en el estado de la cuestión en relación con la visión y la experiencia sobre lactancia materna de las mujeres encarceladas en el Estado español, con un énfasis especial en las situaciones de violencia obstétrica en términos generales, que las puedan aquejar, y que interfieran y/o dificulten (impacten negativamente) sobre dicha lactancia materna, habiendo sido esta ya reconocida como un derecho humano.

Así, la investigación al respecto de la lactancia materna en mujeres encarceladas, tal y como presenta este artículo, supone metodológica y fenomenológicamente, entre otras cosas, la primera vez que se ha traspasado las puertas de un centro penitenciario español para entrevistar a madres acerca de sus lactancias, específicamente, y con una mirada feminista cualitativa, recogiéndose sus voces de primera mano, sus narrativas, sus inquietudes singulares; preguntando en primera persona, también, por sus posibles experiencias traumáticas al respecto de la violencia obstétrica.

La historia nos muestra que la configuración y las dinámicas internas de las prisiones de mujeres, tal y como indica Almeda:

…han tenido, y tienen, su propia historia, su propia filosofía, su propia lógica de funcionamiento y su propia fisonomía, porque a lo largo de los siglos ha habido una forma diferente de castigar a los hombres y mujeres que han vulnerado las leyes penales.^15^

El encarcelamiento de mujeres embarazadas y puérperas ha sido problematizado en numerosos estudios, existiendo inconvenientes graves de atención materno-infantil e instalaciones inadecuadas a estos procesos^16^^,^^17^^,^^18^^,^^19^^,^^20^. De hecho, tanto las prisiones, como muchos de los estudios sobre ellas, se configuran bajo la premisa de un género neutro y universal, asumido en realidad de forma tácita e implícita como masculino. Así, las cárceles continúan

…adoptando patrones universales, falsamente masculinos, siguen discriminando y sancionando a las mujeres por ser mujeres […]. En un mecanismo de dominación social que perpetúa, junto a otros, la subordinación social de todas nosotros como mujeres.^21^

El Consejo de Derechos Humanos de Naciones Unidas^22^ reconoció en 2016 la lactancia materna como un *derecho humano* para madres y criaturas, que ha de ser fomentado y protegido en cualquier circunstancia. Así, se impone la necesidad de su comprensión en un sentido de *universalizabilidad* fuerte y con una connotación claramente feminista^23^. Esta vulneración de un derecho tan básico no debería acontecer *ni siquiera* en el caso de que las madres y su prole se encuentren encarceladas^24^, ya que siempre ha de prevalecer el superior interés de la criatura menor.

La lactancia materna constituye, pues, un derecho humano en tanto que configura:

...la forma sin parangón de proporcionar un alimento ideal para el crecimiento y el desarrollo sano de los lactantes; también es parte integrante del proceso reproductivo, con repercusiones importantes en la salud de las madres.^25^

La lactancia supone una parte elemental de los cuidados esenciales que necesita un ser humano recién nacido, como mínimo, durante los primeros meses de vida, y según la propia Organización Mundial de la Salud (OMS), hasta los dos años, como modo de alimento principal. Implica, por ende, una fenomenología biocultural^26^, que transciende el mero acto alimentario y nutricional, hallándose en la base radical de la salud de la especie^23^.

Según organismos internacionales y la evidencia científica contrastada, es posible constatar las ventajas de la lactancia materna a nivel global^27^. Sus múltiples beneficios para criaturas y madres, a corto y largo plazo, a nivel físico y emocional, están fuera de toda controversia, de modo que habríamos de enfatizar, más que las *ventajas* de amamantar, los *riesgos* de no hacerlo, a una escala planetaria y antropocénica, ya que la práctica lactante supone la forma fisiológica misma de constituirse el ser humano en la exterogestación. Tanto es así, que ya es posible cuantificar estos riesgos/ventajas mediante la herramienta *Cost of not breastfeeding tool*^23^, que tiene como objetivo proporcionar -a los responsables de políticas y defensores- un instrumento que permita hacer estimaciones de los beneficios potenciales para la salud, el capital humano y la economía, al ampliar las estrategias de promoción y apoyo a la lactancia materna. La producción de leche tras el parto es, de facto, un proceso fisiológico (la lactogénesis comienza ya en el embarazo), considerado una parte de la vida sexo-reproductiva de las madres, siempre influido por lo sociocultural y antropológico de manera esencial^23^. 

Por otro lado, la violencia obstétrica ha sido reconocida por la OMS^28^ como:

…una forma específica de violencia ejercida por profesionales de la salud, predominantemente médicos y personal de enfermería, hacia mujeres embarazadas en labor de parto y en puerperio, y constituye una violación a los derechos reproductivos y sexuales de las mujeres.^28^

Sadler^29^ señala que, “si bien […] no ha sido integrado ni reconocido en muchos casos por gremios médicos, sí está siendo ampliamente utilizado por organizaciones de mujeres que buscan instalarlo en la agenda pública”. Además, cada vez hay más evidencia de que se trata de un fenómeno real y contrastable: existen ya incluso varias condenas a España por parte de Naciones Unidas^30^ por prácticas de este tipo de violencia contra diversas madres, y ha sido ya definido como:

…una violación de los derechos humanos, de los derechos a la salud y de los derechos a la salud reproductiva. Tiene que ver con el respeto de los procesos de parto, de los cuerpos de las mujeres, de los tiempos, de la privacidad, de la integridad, de la autonomía y libertad de elección, y de todos los derechos.^31^

En función de todo lo antedicho, la investigación que da origen a este artículo tiene como *objetivo principal* estudiar la experiencia en relación con la lactancia materna de las madres encarceladas en el sistema penitenciario español, con interés en la posible percepción de praxis relacionadas con la violencia obstétrica durante el embarazo, parto y puerperio, asociadas a las dificultades de la práctica lactante. 

El interés motor es contribuir al corpus de conocimientos sobre la cuestión destinado a su aplicación práctica para la mejora de las políticas públicas, en relación con la salud materno-infantil en población privada de libertad. Desde la perspectiva de la interseccionalidad^32^, entendemos la necesidad y la importancia de abordar estas cuestiones en un entorno carcelario, ya que tanto las dificultades típicas de la práctica lactante como las posibles ocurrencias de violencia obstétrica, seguramente se agraven en un entorno ya de por sí de máxima vulnerabilidad a diferentes formas de discriminación.

### Marco epistémico-conceptual: cárceles, mujeres y madres, en la discriminación interseccional

Pese a la existencia de leyes penitenciarias que detallan la posibilidad de que las mujeres recluidas

...pudieran estar embarazadas o tener hijos lactantes, las regulaciones son escasísimas, comparadas con la cantidad de artículos dedicados a otras cuestiones de la vida en prisión (por ejemplo, el trabajo penitenciario, organización y gestión de economatos, etc.).^33^

En este sentido, Baldwin^34^ afirma que existe una comprensión limitada de las necesidades en la maternidad por parte de las instituciones correccionales y los proveedores de servicios de maternidad, así como grandes carencias en la respuesta a estas necesidades. 

Por otro lado, recientes investigaciones reflexionan en torno a la situación de bienestar de las criaturas que se encuentran encarceladas junto a sus madres, preguntándose si las desigualdades que la infancia padece debido al encarcelamiento son justificables desde una perspectiva de justicia social^35^. Por ello, son de vital importancia unas políticas de salud materno-infantil que promuevan la atención de salud cualificada y segura, especialmente en relación con los derechos reproductivos, tales como el apoyo a la nutrición, el cuidado diario y, sobre todo, la lactancia^9^^,^^10^.

Si acudimos a la historia sobre las cárceles en sí mismas, encontramos que la literatura existente las muestra como estructuras punitivas relativamente recientes, ya que hasta finales del siglo XVIII no empiezan a existir instituciones en las que someter a un régimen de aislamiento y privación de libertad, como tal, a personas condenadas^36^; aunque conviene destacar que ya existe una corriente historiográfica que plantea las raíces de la privación de libertad en las prácticas de la ortodoxia cristiana del siglo XIII^37^.

Según datos del último Anuario Estadístico^38^, correspondiente a 2021 y publicado por el Ministerio del Interior de España, al 31 de diciembre de dicho año había 55.097 personas encarceladas en las prisiones del país. De estas, el 92,9% eran hombres y el 7,1%, mujeres. De los cerca de 80 centros penitenciarios estatales, solo cuatro de ellos son cárceles para mujeres (Wad-Ras, en Barcelona; Alcalá de Guadaíra, en Sevilla; Madrid-I, en Madrid, y Brieva, en Ávila). Esto implica que, a pesar de que la Ley Orgánica 1/1979^39^ establece las prisiones *exclusivas* para mujeres, en la práctica, ellas deben cumplir condena en módulos femeninos dentro de cárceles de hombres.

La historia nos muestra que la configuración y las dinámicas internas de las instituciones de reclusión femenina,

...han tenido, y tienen, su propia historia, su propia filosofía, su propia lógica de funcionamiento y su propia fisonomía, porque a lo largo de los siglos ha habido una forma diferente de castigar a los hombres y mujeres que han vulnerado las leyes penales.^15^

De esta forma, las mujeres están expuestas a una múltiple condena: la personal (por el distanciamiento con su familia y por dejar de cumplir con el rol tradicionalmente asignado de “madre, hija, esposa cuidadora”), la social (por haber transgredido las normas socialmente esperadas por ser mujer), y la que hace referencia al delito en sí (teniendo que asumir condiciones más duras que los hombres durante el cumplimiento de la condena), todo ello viéndose agravado en el caso de ser madres, según el informe de 2021 de la Asociación Proderechos Humanos de Andalucía^40^.

Aguilera y Martínez^41^ indican que el 80% de las mujeres en prisión son madres. Hasta la reforma de la Ley Orgánica General Penitenciaria de 1996, las madres podían estar en la cárcel junto a su prole hasta los seis años. Sin embargo, tras dicha modificación solo se permite que las criaturas estén en los centros penitenciarios hasta los tres años. De este porcentaje, tan solo una minoría, y siempre que la edad de las criaturas y la situación penal de las madres lo permita, conviven sus hijos e hijas intramuros en las llamadas Unidades de Madres. Existen diferentes tipos de Unidades de Madres: internas (pabellones dentro de cárceles), externas (edificios anexos a la prisión), mixtas (bloques donde ambos progenitores cumplen condena), y dependientes (centros para madres que se encuentran en régimen de semilibertad) (Tabla 1).

**Tabla1. Número:** de madres y criaturas encarceladas, según las unidades de madres del sistema penitenciario español. 2022.

Administración	Tipo de unidad de madres	Ubicación	Número de madres (n=79)	Número de criaturas (n=82)
Secretaría General de Instituciones Penitenciarias, Ministerio del Interior	Interna	Madrid VI (Aranjuez)	24	24
	Externa	Mallorca	1	1
	Externa	Madrid	5	5
	Externa	Sevilla	10	11
	Externa	Alicante	16	16
	Mixta	Madrid VI (Aranjuez)	12	12
	Dependiente	Madrid VI (Aranjuez)	4	5
Departament de Justícia de Catalunya	Interna	Barcelona (Cárcel de Wad-Ras)	7	8
Departamento de Igualdad, Justicia y Políticas Sociales de País Vasco	Interna	Donostia (Cárcel de Martutene)	0	0

Fuente: elaboración propia.

## MÉTODO

Se realizó un estudio exploratorio-descriptivo con abordaje cualitativo, en cuatro instituciones ubicadas en cuatro localidades diferentes del territorio español, a saber: la Unidad de Madres Externa (UME) de Alicante, Madrid y Sevilla y el Departamento de Madres (DM) en el interior de la Cárcel de Wad-Ras, en Barcelona. El periodo de investigación de campo abarca desde diciembre de 2021 hasta abril de 2022.

Desde 1983, el Estado español cuenta con una Administración Penitenciaria Central para todas las instituciones penitenciarias de su territorio nacional, excepto en Cataluña, que es el Departamento de Justicia el que tiene competencias en materia penitenciaria. Así mismo, País Vasco (desde mayo de 2021) asume la gestión penitenciaria de los centros de su territorio.

La población de estudio la conformaron 30 de las mujeres mayores de edad que se encontraban cumpliendo condena en los diferentes centros junto a sus criaturas. El acercamiento inicial se realizó a través de la dirección de cada Unidad de Madres, la investigadora que desarrolló el trabajo de campo les facilitó información sobre la investigación y la dirección les propuso participar de forma voluntaria. Un total de 30 madres aceptó participar en el estudio. Por las mismas vías se establecieron las fechas para llevar a cabo el trabajo de campo. Para su realización, se solicitó y se obtuvo la autorización de la Secretaría General de Instituciones Penitenciarias y el Departamento de Justicia de Cataluña. Y tal y como es preceptivo, se contó con el consentimiento informado escrito de todas las personas que fueron entrevistadas. Desde el proyecto original de esta investigación, el diseño del plan de trabajo contó con la supervisión y la aprobación correspondientes en el marco legal del Programa de Doctorado y la institución superior a los que pertenece (Universidad de Granada), incluyendo lo relativo a la revisión y la aprobación de los aspectos éticos.

Se optó por el método etnográfico con perspectiva *crítica,* la cual

...presenta diferencias fundamentales con la etnografía tradicional y es de gran relevancia debido a las fuertes críticas sociales y políticas que promueve, lo cual repercute en mejoras para la comunidad en estudio.^42^

En congruencia con el carácter y las temáticas de la investigación, relacionadas a entornos complejos de máxima vulnerabilidad y población (interlocutores), en situaciones de discriminación interseccional.

Las técnicas primordiales fueron la entrevista semiestructurada^43^ y la observación participante^44^. La entrevista semiestructurada presenta un grado mayor de flexibilidad que las estructuradas o cuestionarios al uso, con el fin de ajustarse a las interlocutoras y partiendo de un guion previo flexible; este se fue modificando ligeramente a medida que avanzaba la recolección de datos, en aras de la necesaria adaptabilidad de un proceso de investigación de carácter cualitativo, y en un entorno tan desafiante como es el ámbito carcelario. Finalmente, la observación participante se practicó de manera paradigmática y estructural, lográndose información de las actividades cotidianas e interacciones de las madres en su entorno habitual.

Todas las entrevistas se llevaron a cabo en un espacio privado para proteger la confidencialidad, y variaron de 15 a 70 minutos de duración, con una media de 31 minutos. Se mantuvieron hasta que se alcanzó el punto de saturación. Se grabaron en soporte digital y se transcribieron de forma literal. Se analizaron según la teoría fundamentada^45^, utilizándose el software de datos cualitativos MAXQDA, así como el llamado *método de comparación constante*, que pretende “fundamentar los conceptos en los datos […] y, para ello, se requiere como ingrediente fundamental la creatividad y el pensamiento crítico de los investigadores”^46^. Como resultado de este proceso, se obtuvieron las siguientes categorías emergentes: 1) motivación para amamantar y percepciones de la agencia lactante, 2) dificultades e interferencias percibidas durante la lactancia materna, y/o en relación con su fomento y apoyo en los sistemas sanitario y penitenciario, 3) aculturación lactante y falsas creencias sobre la lactancia materna, y 4) situaciones que aluden a la violencia obstétrica.

Al tratarse de una investigación con perspectiva de género y feminista, se empleó el marco teórico de la interseccionalidad^32^, “el tropo feminista más difundido para hablar ya sea de identidades o de desigualdades múltiples e interdependientes”^47^, y teniendo en cuenta aquellos postulados que ponen en valor la epistemología de las emociones en los procesos de investigación^48^.

## RESULTADOS Y DISCUSIÓN

Se presentan los resultados junto a la discusión, ya que el diálogo con la bibliografía precedente sobre el tema, por un lado, y con las perfectivas feministas y de la interseccionalidad, por otro lado, se entreteje con los resultados de manera dinámica y sistemática.

### Datos sociodemográficos: edad, procedencia, estudios/vida laboral

La población de estudio (Tabla 2) la conformaron 30 de las mujeres mayores de edad que se encontraban cumpliendo condena junto a sus criaturas en los diferentes centros. Tenían entre 19 y 45 años. El 53% de las mujeres en las Unidades de Madres están en el rango 31-40 años, seguidas por las del rango de edad 18-30; es decir, la mayoría se encuentran entre los 18 y 40 años, lo que podría considerarse como período reproductivo, en términos generales.

**Tabla 2:** Datos sociodemográficos de las madres entrevistadas (n= 30), según las unidades de madres del sistema penitenciario español. Diciembre 2021 - abril 2022.

Datos sociodemográficos	UME Madrid (n= 4)	UME Alicante (n= 14)	UME Sevilla (n= 6)	DM de Barcelona (n= 6)
Edad de las madres				
18 a 30	4	3	1	2
31 a 40	-	8	4	4
41 a 50	-	2	1	-
NC	-	1	-	-
País de procedencia				
Argelia	-	1	-	-
Bolivia	-	1	-	-
Brasil	-	1	-	-
Chile	-	-	-	1
Colombia	-	1	-	-
Costa Rica	-	-	-	1
España	1	7	5	3
Italia	-	1	-	-
Nigeria	-	1	-	-
Perú	2	-	1	1
Rusia	-	1	-	-
Venezuela	1	-	-	-
Escolaridad				
Primaria	-	10	5	3
Secundaria	4	2	-	2
Universitaria	-	1	1	-
NC	-	1	-	1
Ocupación antes de entrar en prisión				
Contables, administrativas	2	-	-	-
Empresaria	-	2	-	-
Estudiantes	-	-	-	-
Gerocultoras	-	-	1	1
Personal de limpieza	1	2	2	1
Servicios de hotelería y vendedoras	1	4	1	2
Sin trabajo remunerado	-	6	2	-
NC	-	-	-	2

Fuente: elaboración propia. UME= Unidad de Madres Externa. DM= Departamento de Madres. NC= No contesta.

Las madres provenían de África, Europa, Europa del Este y Latinoamérica. El 53% de ellas eran de origen español. Esto puede deberse al hecho de que en las Unidades de Madres la mayoría de las mujeres que cumplen condenas se encuentran con medidas que permiten flexibilizar la clasificación penitenciaria, como señala el Artículo 100.2 del Reglamento Penitenciario o incluso de tercer grado, es decir, cumpliendo una pena privativa de libertad, pero llevando a cabo un régimen de vida en semilibertad (excepto en la Unidad de Madres de Alicante, donde hay internas preventivas, penadas de larga duración, con delitos graves o muy graves, con ciertos comportamientos desadaptativos o alteraciones mentales significativas). Por lo tanto, es posible que se hayan adoptado medidas alternativas, como la expulsión en caso de mujeres extranjeras, según criterios de la Ley Orgánica 10 de 1995 del Código Penal, Artículo 89 del 23 de noviembre de 1995^49^, antes de que puedan llegar a cumplir su condena en la Unidad de Madres.

Con relación al nivel de escolaridad alcanzado, la mayoría tenía estudios primarios (18 de ellas), seguido de secundarios (8 de ellas) y universitarios (2 de ellas). A nivel profesional, el 33% de las madres trabajaba en el sector de hotelería, el 20% era amas de casa y el 16% era personal de limpieza. Siguiendo a Yagüe^50^, el análisis del perfil socioeconómico de las mujeres encarceladas muestra que estas ya provienen de las capas más desfavorecidas de la sociedad, y con un historial evidente de discriminación y exclusión a sus espaldas: familias desestructuradas (en el umbral de la pobreza), problemáticas de drogodependencias, una escasa o nula formación y profesionalización, víctimas de violencia machista, con fuertes cargas familiares, entre otros factores. Todo ello, además de favorecer que las mujeres cometan algún delito, dificulta *a posteriori* una posible reinserción una vez cumplida la condena, abocándolas de esta forma a una espiral de pobreza y marginalidad. Es decir, la prisión “excluye a mujeres que no estaban excluidas antes de estar en prisión y agrava la marginación de las que ya lo estaban”^33^.

Los datos recogidos en la Tabla 3 muestran que un total de 30 madres cumplía condena junto a un hijo o hija, y una de ellas, además, estaba embarazada. Un par de madres tenía 2 hijos o hijas en prisión. Es fundamental señalar que estas madres, además de estar encarceladas junto a una o varias criaturas, tienen responsabilidades familiares en el exterior: un 43% tenía más de 2 hijos o hijas extramuros, seguido de un 20% con 1 o 2 hijos o hijas, y un 13% sin tener más hijos o hijas fuera de la cárcel. El hecho de que existan pocas Unidades de Madres, hace que se dé una evidente dispersión y, por consiguiente, una mayor dificultad para poder mantener el contacto con sus hijos e hijas que no se encuentran intramuros, e “inevitablemente las mujeres tienen que sostener la pérdida de arraigo a causa de la separación de sus hijos e hijas”^33^.

**Tabla 3:** Situación de embarazo, número de hijos e hijas en prisión y edad, y número de hijos e hijas fuera de prisión de las madres entrevistadas (n= 30), según unidades de madres del sistema penitenciario español. Diciembre 2021 - abril 2022.

	UME Madrid (n= 4)	UME Alicante (n= 14)	UME Sevilla (n= 6)	DM de Barcelona (n= 6)
Situación de embarazo				
Con hijos/hijas sin embarazo	4	14	6	6
Con hijos/hijas y embarazadas	-	1	-	-
Hijos/hijas en prisión				
1 hijo/hija	4	14	5	5
2 hijos/hijas	-	-	1	1
Edad hijos/hijas en prisión (en meses)*				
0 a 6	-	4	1	2
7 a 12	2	1	2	1
13 a 24	1	5	1	1
25 a 36	1	3	1	2
37 y más	-	-	1	-
NC	-	1	-	1
Hijos/hijas fuera de prisión				
0 hijo/hija	2	2	-	-
1 hijo/hija	1	1	1	2
2 hijos/hijas	1	4	1	1
2 hijos/hijas y más	-	7	4	2
NC	-	-	-	1

Fuente: elaboración propia. *Se indica el número de niños y niñas que están en prisión junto a sus madres, y no el número de madres que tienen criaturas en esas franjas etarias, por lo que el número total es mayor al número de madres. UME= Unidad de Madres Externa. DM= Departamento de Madres. NC= No contesta.

### Datos específicos en relación con la lactancia materna

En la Tabla 4 y en relación con la lactancia materna, el 77% afirmó haber dado el pecho a sus hijos e hijas estando en la cárcel. De las 23 madres que amamantaron a sus criaturas, más del 50% optó por la lactancia materna exclusiva y el resto por la lactancia materna mixta. En cuanto al tiempo de duración, nueve de ellas mantuvieron la lactancia de 0 a 3 meses; cinco de las madres, de 4 a 6 meses; ninguna en la franja de 7 a 24 meses; tan solo una de las madres superó los 24 meses, y el resto seguía dando el pecho en el momento de realizar la entrevista.

**Tabla 4:** Número de madres entrevistadas que amantaron, tipo de lactancia y duración, según unidades de madres del sistema penitenciario español. Diciembre 2021 - abril 2022.

	UME Madrid (n= 4)	UME Alicante (n= 14)	UME Sevilla (n= 6)	DM de Barcelona (n= 6)
Amamantaron a sus hijos/hijas en prisión				
Sí	3	10	6	4
No	1	4	-	1
NC	-	-	-	1
Tipo de lactancia				
Exclusiva	1	6	2	4
Mixta	2	4	4	-
Duración (en meses)				
0 a 3	2	4	1	2
4 a 6	-	2	2	1
7 a 12	-	-	-	-
13 a 24	-	-	-	-
25 y más	-	1	-	-
No ha finalizado	1	3	3	1

Fuente: elaboración propia. UME= Unidad de Madres Externa. DM= Departamento de Madres. NC= No contesta.

Como es habitual en estudios antropológicos-etnográficos, se irán presentando los hallazgos y categorías a la par de los propios términos, expresiones y narrativas de las interlocutoras, habiéndose seleccionado algunos de los *verbatim* más representativos o significativos, respetándose siempre, como es propio, las palabras y expresiones orales originales.

### Categorías emergentes

#### Motivación para amamantar y percepciones de la agencia lactante

En congruencia con lo presentado en la primera parte del artículo, la mayoría de las madres reconocía y ponía en valor la importancia de amamantar a sus hijos e hijas, pero emergen de sus relatos diferentes enfoques, relacionados con motivaciones extrínsecas o intrínsecas.

Algunas de ellas manifestaban el deseo de experimentar en primera persona lo que significa el acto de amamantar y la vivencia de estar piel con piel:

*Me encanta. Yo a él no le iba a dar porque como llevaba tanto* [sin dar el pecho], *mi hija por ejemplo ya es más mayor. Pero es que el tenerlos ahí me encanta*. (Madre 10)

Otras consideraban la lactancia materna como la mejor forma de alimentación para un sano crecimiento de sus bebés:

*Inconscientemente, como yo soy madre, sé que la lactancia materna es lo mejor que puede haber para el estómago del bebé y yo siempre les daba a mis hijos pecho*. (Madre 3)

También aludían a la lactancia materna como gran facilitador de la instauración y mantenimiento del vínculo en la díada madre-bebé:

*Es muy bonito para mí. Yo pienso… yo respeto a todo el mundo y que cada persona haga lo que quiera, pero yo pienso que los niños… lo necesitan. Es bueno…, es un vínculo que creas con tu hijo. Y a ella le encanta, vamos*. (Madre 2)

Esto último puede resultar de vital importancia en un contexto donde la posibilidad de separación y el miedo a que ocurra está siempre presente; así, la lactancia materna estaría contribuyendo a generar lo que Bronfenbrenner^51^ denomina un ecosistema sano, que favorece un apego saludable^52^^,^^53^.

Por otro lado, el hecho de amamantar resulta en muchos casos un acto de empoderamiento, en tanto que hay una reapropiación y “agencia sobre el propio cuerpo”^26^. De forma análoga ocurre intramuros, pudiendo ejercer la lactancia materna un efecto positivo en las madres encarceladas, pues en este contexto hay una expropiación sistemática del poder de decisión (“*hasta para llamar a mi familia, ellos deciden cuándo*”) y brinda la posibilidad de ocupar un espacio libremente (“*yo le doy la teta por cualquier lado de la cárcel*”), ya que la prisión es un espacio en el que se imponen continuamente límites y prohibiciones. Ello supondría un factor potencialmente protector, pues la prisión es limitante para la vida de las personas presas y también para el propio cuerpo, con graves consecuencias a nivel físico, mental, emocional y social^54^.

Muchas de las madres manifestaron el deseo de haber podido transitar esta experiencia, aunque en ocasiones no lo hubieran logrado:

*A mí me hubiera gustado darle más tiempo. Porque yo veo una madre que le da el pecho a su niño y a mí eso me da ilusión la verdad. Pero a mí no me ha tocado*. (Madre 23)

Y, además, el hecho de no haber podido amamantar a sus criaturas les generaba sentimientos de intensa tristeza:

*Hombre, me dio pena. Yo lloré un montón cuando me dijo que mi leche no valía y no cogía peso el niño. Porque yo quería darle nada más que pecho. Quería intentarlo y no pude*. (Madre 20)

E incluso hacen alusión a que ha sido un trauma para ellas:

*Digo: tendrías que haberlo hecho, tendrías que haber seguido. Aquí hay chicas que sacan el pecho y lo hacen tan tranquilas, están habituadas a la cárcel, yo no. Para mí es un trauma* [no haber podido]. (Madre 9)

También emergieron sentimientos de culpa al no conseguir amamantar a sus criaturas, lo cual puede repercutir negativamente en la relación con el bebé y/o en la autoestima materna, y se trata de un tema largamente tratado en la promoción crítica de lactancia^55^.

*Cuando llegué lo intenté y sí que tomaba, fue culpa mía porque no me esforcé. Me siento mal*. […] *a mi hija le cuesta comer. Está bien de peso, en la media y todo, pero me ha dolido porque yo los he criado muy bien así. Después ya le daba sus pucheritos, la pasta nuestra… y ver que no puedo hacer eso con mi hija, duele, me duele mucho*. (Madre 9)

Además, manifestaron conocimiento de que durante el acto de amamantar se crea un fuerte vínculo y que ello es muy positivo para construir un apego saludable entre madre y bebé^52^^,^^53^.

*Es muy bonito para mí. Yo pienso… yo respeto a todo el mundo y que cada persona haga lo que quiera, pero yo pienso que a los niños…, lo necesitan. Es bueno…, es un vínculo que creas con tu hijo. Y a ella le encanta, vamos.* (Madre 2)

#### Dificultades e interferencias percibidas durante la lactancia materna, y/o en relación con su fomento y apoyo en los sistemas sanitario y penitenciario

Es sabido que, en la mayoría de las ocasiones en las que no se da una lactancia materna exitosa, los motivos poco tienen que ver directamente con el comportamiento o las acciones de las madres sino, más bien, con las diversas interferencias y faltas de apoyo adecuado que acontecen en el entorno directo. En el caso de las madres en prisión, esto resulta especialmente relevante, ya que se encuentran en una situación de extrema vulnerabilidad, y en condiciones mucho más desventajosas frente a madres que atraviesan esta experiencia en libertad.

Algunas de las dificultades que expresan/experimentan tienen que ver con la posibilidad de que la díada madre-bebé sea separada en un determinado momento; por ejemplo, a causa de la detención:

*No le di el pecho porque yo la he dejado con un mes y me la han traído la niña con 3 meses*. (Madre 13)

Otra de las barreras que pueden encontrarse las madres durante este período es que los profesionales médicos recomienden dejar la lactancia materna bajo el pretexto de administrar ciertos medicamentos, de forma análoga a lo que ocurre extramuros.

En ocasiones, estas recomendaciones no están basadas en evidencia científica actualizada, y el “desconocimiento de los beneficios de la lactancia y de los estudios recientes sobre la farmacocinética en la lactancia motiva que con frecuencia los psiquiatras o los médicos de atención primaria recomienden el destete temprano para que la madre pueda iniciar tratamiento psicofarmacológico”^56^.

*Yo quería seguir. Pero mira, es más importante que me salga* [la placenta] *y no me quede nada dentro. Es que no paro desde ayer que me lo han dicho…, es que me esperaba una buena noticia, mira, ya no lo tienes. Y que me hayan dicho esto me ha asustado. Que me podrían intervenir, otra vez abrirme por ahí. Es un rollo*. […] *No se puede. Así me han dicho. Yo: ¿tengo que dejar la leche? Sí*. (Madre 5)

La salud mental de las madres se ve comprometida a causa de múltiples factores que concurren, precisamente, en personas encarceladas, que son más susceptibles a ello: “los embarazos a menudo no son planificados y se complican por la falta de atención prenatal, el trauma materno, la mala nutrición, el consumo de sustancias, las enfermedades mentales, las afecciones médicas crónicas, el bajo nivel socioeconómico y el apoyo social limitado”^20^. El conjunto de todo ello hace que, a menudo, estas madres precisen de tratamiento psicofarmacológico.

Existe evidencia contrastada de que algunos antidepresivos, ansiolíticos e incluso el carbonato de litio, si se utiliza con prudencia, son compatibles con la lactancia materna^56^, según se indica en *e-lactancia,* un proyecto de la Asociación para la Promoción e Investigación Científica y Cultural de la Lactancia Materna (APILAM).

Con todo, todavía existen profesionales sanitarios que aconsejan abandonar la lactancia materna sin un motivo realmente justificado, vulnerando la premisa de que “los beneficios de la lactancia materna exceden ampliamente a los de la lactancia artificial, por lo que los profesionales de la salud deberían tener muy presentes dichos beneficios y favorecer la continuación la lactancia siempre que sea posible”^56^.

Con relación a la salud mental de las madres, es cierto que una de las Unidades de Madres cuenta con un proyecto de salud mental perinatal, un programa que aborda los trastornos mentales que tienen las mujeres durante el embarazo y/o los primeros 12 meses posparto. Sin embargo, encontramos que las actuaciones posiblemente no resultaron oportunas o significativas para la población diana de forma óptima, en tanto que las madres de esta Unidad no *reconocían* las intervenciones efectuadas y manifestaban haber sentido falta de apoyo psicológico durante la etapa perinatal.

Es muy probable que la extrema dureza de las condiciones de hallarse en privación de libertad, así como el ambiente hostil que se experimenta dentro de la cárcel, devengan en interferencias específicas para con la lactancia materna exclusiva, dado que se trata un proceso complejo y delicado, que *necesita* que la madre *sienta* que puede, además de desearlo; en muchos casos, la indefensión aprendida es lo habitual:

*Con mi hija no pude porque, te voy a ser muy sincera, primero porque yo no me sentía bien. Yo no estaba bien psicológicamente. Estaba asumiendo algo muy duro. No me sentía tranquila, estaba insegura de todo. El ambiente de aquí…, todo eso porque no deja de ser una cárcel*. […] *Tienes que estar siempre con tu coraza. Si tienes que estar en tantas cosas y encima sacarte tus defensas para luchar fuera también, con todo lo que tenía… no me vi capacitada*. (Madre 9)

Por otro lado, algunas de las madres manifiestan haber tenido graves dificultades a causa del dolor físico que sentía mientras amamantaban a sus criaturas, debido a un agarre incorrecto, a una posición poco efectiva, etc. La evidencia muestra que hay una asociación entre problemas tempranos y una mala técnica, con lo que la “técnica de amamantamiento correcto constituye la base de una lactancia exitosa y de prevención de problemas mamarios asociados a la lactancia materna como grietas en el pezón, ingurgitación mamaria o mastitis”^57^.

*Es que es dolor que ya he pasado las dos semanas críticas de tener sangre, la subida… Mira, si tengo hasta esto que me dieron ahí en el hospital* [pezoneras]. *Porque es que me moría*. (Madre 5)

Referente a la percepción de haber tenido ayuda antes las posibles dificultades durante su experiencia, de las narraciones se extrae que, en la mayoría de las ocasiones, no existe una gran sensación de haber contado con apoyo efectivo en las Unidades de Madres por parte del personal, sea sanitario o no. A pesar de la suma importancia que esta asistencia entraña para la práctica lactante, no siempre existen estrategias de promoción, protección y apoyo en las cárceles (ni fuera tampoco), que consigan acompañar de forma correcta y significativa a las madres encarceladas^9^, lo cual explicaría en gran medida la hipogalactia social global que ha sido analizada y descrita, fruto de una aculturación social masiva en torno a la lactancia^23^:

*Yo sí quería dar el pecho, pero fue muy complicado porque yo no tenía persona que me ayudara*. (Madre 14)

*Nadie. No pude hablar con nadie. Todo biberón y toma leche y ya está. Se acabó el problema*. (Madre 22)

También se detecta una falta de apoyo importante cuando las madres se encontraban en el hospital tras dar a luz:

*Me hubiera encantado que alguien me dijera: “tranquila, vamos con un sacaleches. Vamos a empezar aquí otra vez y vas a ver como no pasa nada”. Además F. nació súper chiquitín, mucho miedo con él*. (Madre 22)

Muchas de las madres relatan haber pedido ayuda al personal sanitario, tanto dentro como fuera de prisión, y que este haya optado por recomendar leche de fórmula en lugar de fomentar la lactancia materna:

*Aquí te dan leche y el biberón, te dan todo. Porque el médico* [de la Unidad de Madres] *me ha dicho que ella se quedaba siempre con hambre*. (Madre 8)

No obstante, de algunos relatos sí se extrae que en contadas ocasiones las madres han encontrado profesionales sanitarios (del mismo centro penitenciario, o bien matronas externas) que han fomentado la lactancia materna y las han apoyado ante dificultades:

*Ellas vienen aquí a hacer charlas. Hace cosa de 10 o 15 días nos vinieron a dar una charla, la matrona, la misma que nos atiende, sobre qué son los anticonceptivos, de la lactancia y todo eso*. (Madre 29)

En sus “*herstories*”^58^ -término feminista resultante de un juego de palabras que literalmente sería “el relato *de ella*”, ya empleado por Massó^2^^,^^59^, a modo de reconstrucción de historias de vida lactantes/lactivistas- queda patente que estas madres suelen acudir a sus compañeras (sus pares) cuando tienen alguna duda o problema durante la lactancia materna, incluso antes de hablar con los profesionales. Esto se debe a que confían en la experiencia de las *otras* (sus pares, sus iguales) que han sido madres antes, porque se sienten más cómodas compartiendo su inquietud con una figura femenina similar, operando bajo la misma lógica que el *lactivismo* (activismo prolactancia), que “muestra una combinación singularísima de los ámbitos duales clásicos privado-público o naturaleza-cultura, ejerciendo como política transformadora de cuerpos, costumbres, sociedades”^2^:

*Entre nosotras nos preguntamos. Les digo: “ponte un poquito de crema o ponte cualquier cosa”. Sí, aquí entre nosotras nos ayudamos*. (Madre 12)

Incluso, hay discursos explícitos sobre el género de las profesionales sanitarias y cuáles son las fuentes de confianza preferidas:

*Había una pediatra, pero es para los niños y no para nosotras. Y cuando tienes que hacer una consulta, decía, mira: “los jueves cada 15 días venía el medico”. Pues no era normal, hablar con una médica, que un médico. No me sentía igual. Entonces yo decidía no hablar*. (Madre 10).

Todo ello muestra que todavía hoy en día no hay personal sanitario (o no el suficiente) formado y actualizado en cuanto a lactancia materna se refiere, a pesar de que numerosos estudios (biológicos y epidemiológicos) evidencian que la decisión de no amamantar presenta importantes efectos negativos sobre la nutrición, el desarrollo y la salud del bebé y de la madre (como señalamos al inicio). Esto condiciona que la alimentación con leche materna sea la intervención sanitaria que, con menores costos económicos, consigue mayores beneficios sobre la salud^60^; o, en otras palabras, la práctica sanitaria con relación costo-beneficio más ventajosa^23^.

#### Aculturación lactante y las falsas creencias sobre la lactancia materna

Muchas de las narrativas recogen la reproducción o asimilación de algunas de las falsas creencias sobre lactancia materna que se generalizaron en la cultura occidental especialmente a partir de la segunda mitad del siglo XX, y como herencia de la llamada “cultura del biberón”; lo que, en otros términos, podríamos llamar una auténtica aculturación lactante^61^, que ha generado la pandemia de hipogalactia social a nivel global^23^, a tenor de las cifras tan preocupantemente bajas sobre lactancia^62^.

Hablamos de falsas creencias^63^ que se han transmitido cultural y socialmente (una socialización lactante errónea, deletérea), así como de opiniones muy arraigadas, que contribuyen a que se pierda la cultura del amamantamiento y una correcta socialización en esta práctica biocultural. Por otro lado, estas creencias falaces han sido ya largamente revisadas y contraargumentadas, incluso desde la fenomenología feminista^2^. A menudo, provienen del propio personal sanitario (dentro y fuera de prisión), del personal penitenciario (educadoras, funcionarias, etc.) y, por supuesto, de las mismas presas, y todo ello puede devenir en interferencias en la instauración o el mantenimiento de la lactancia materna, con graves consecuencias en sus experiencias. Por ejemplo, las que hacen referencia a “*escasez de leche*” o “*poca producción de leche*”:

*Yo le dije a la pediatra* [del hospital] *que él no se llenaba con mi pecho y que no me salía mucha leche, y me dijo que tomara mucho líquido y que le diera de mi pecho y biberón. Le estuve dando pecho y biberón, pero se llenaba de gases y le dije a la pediatra y me dijo: “escoge, una de las dos porque no te sale mucha leche… y si ya le estás dando fórmula, sigue dándole fórmula mejor”. Y al mes y medio ya le daba solo fórmula*. (Madre 3)

*Cuando le daba el pecho a la niña, no se me llenaba y a mí me dolía mucho el pecho* [...]. *Me dijeron que soy de poca producción de leche*. (Madre 25)

Encontramos referencias también a la forma o tamaño “incorrecto” de la mama (siendo ya evidenciado científicamente^64^ que no hay cánones fisiológicos en este sentido: cualquier pecho es apto para amamantar, salvo porcentajes mínimos vinculados a ciertas morbilidades, y sin significación estadística alguna):

*A mí me gustó. Si hubiera podido, hubiera durado más... pero con ese problema de la teta grande y una pequeña, yo no aguanté y lo paré*. (Madre 15)

Hallamos alusiones relacionadas con la supuesta “*mala calidad*” de la leche materna y a las “*malas tetas*”:

*Estuve dándole solamente el pecho un mes porque no quería darle leche* [fórmula]. *Y al mes cuando me hicieron el control, vieron que el niño no cogía peso. Entonces me dijo la pediatra* [de la Unidad de Madres] que tenía que darle una ayuda porque se ve que mi leche no le llenaba. (Madre 20)

*Estuve un mes dándole. No le di más porque tenía mala teta. No me salía leche. El niño se me moría de hambre, lloraba mucho* […] y *le enchufé el biberón*. (Madre 26)

Recogimos también relatos sobre la lactancia prolongada^65^, es decir, la creencia de que, con cierta edad de la criatura, ya no procede y está mal visto amamantarla:

*A mí las educadoras me dijeron que, teniendo la edad que tenía, ya no era conveniente que siguiera dándole pecho porque lo que deberíamos hacer es reforzarlo,* […] *como que no lo hacemos mayor. Que si le das pecho sigue siendo bebé, que no le dejamos avanzar mentalmente*. (Madre 21)

Con respecto a la creencia arraigada de que dar el pecho es doloroso y que sentir ese dolor es “*normal*”, algunas madres afirmaron:

*Es que las grietas nos salen a todas. Después se te pasa*. (Madre 12)

#### Situaciones que hacen alusión a la violencia obstétrica

De los relatos analizados también se extrae que algunas de las madres han pasado por momentos en los que se ha ejercido violencia obstétrica contra ellas y/o sus criaturas; cuestión, como mencionamos, vinculada de forma estructural con la práctica lactante, ya que impacta directa y negativamente con su correcta instauración.

Las madres refieren haberla padecido durante toda la etapa perinatal, por parte del personal sanitario, tanto del centro penitenciario como del hospitalario, ya fuera en forma de acción u omisión, y evidenciando graves consecuencias para su salud:

*Y nunca se me va a olvidar que no podía parir hasta que no esté el resultado de la PCR. Y yo con esas contracciones que me moría. Y yo nomás me acuerdo que dijeron “PCR negativa”, porque yo llegué de 7 cm de dilatación. Yo rompí aguas y el niño se me bajó. Y yo me lo metía para adentro. Porque me decían que tenía que esperar. Y recuerdo que la matrona dijo: “ahora vengo, si necesitas algo me dices”. Y cuando escuché que la PCR era negativa, apreté el botón rojo y me dice: “bueno, vamos a prepararnos”. Y yo le dije: “que no hay que prepararse nada”. Cogí, abrí mis piernas y ella se quedó parada mirando y yo sola de un solo pujo lo boté. Recuerdo que se le resbalaba. Y me decía: “¿cómo hiciste eso?”. Y yo: “es que no podía más”. Claro, es que no le dio tiempo a ponerse los guantes*. (Madre 1)

Los relatos describen situaciones de maltrato, también, durante el puerperio, siendo una etapa especialmente vulnerable para las madres que acaban de dar a luz, y ello a pesar de que cada vez hay más evidencia científica que muestra que “el estado emocional de la mujer durante embarazo, parto y posparto tiene enormes repercusiones, tanto para ella como para el bebé que gesta, para la salud física y mental de todos y cada uno de los miembros de la familia, a corto y largo plazo”^66^:

*Los policías…, yo estoy en una cama, recién parida. Y ellos están en un sofá. Con el móvil y la tablet a todo volumen, y tú recién parida. Estás sola, no tenés a nadie, y ellos están gritando, riéndose. Horrible. Vas al baño y tardas cinco minutos y ya te están tocando: “¡¿por qué tardas?!”. Pero a ver… ¿por dónde voy a salir…, por el váter?* (Madre 1)

Se conoce que la depresión posparto puede manifestarse entre el 10% y el 15% de las mujeres. Los factores de riesgo de la depresión posparto, según Antúnez *et al*. son:

…factores estresantes de la vida diaria (conflictos de pareja, haber pasado eventos estresantes durante el embarazo, situación económica, estado civil...), falta de apoyo, malos resultados obstétricos previos [...], problemas con la lactancia, edad gestacional, cambios hormonales, privación del sueño y la susceptibilidad genética.^67^

Otros factores de riesgo de la depresión posparto son: parto por cesárea, primiparidad y trastornos de salud mental previos^67^. Hay estudios que muestran que las mujeres que experimentan depresión posparto han pasado por factores de vida estresantes durante el embarazo y después del parto^67^^,^^68^. Algunas de estas circunstancias se encuentran presentes en los relatos de las madres encarceladas y muchas de ellas informaron haber padecido altos niveles de estrés. En este sentido, varias de las madres reportaron haber sentido extrema tristeza e intuir o sospechar que podrían estar sufriendo una depresión posparto, no habiendo sido nunca diagnosticadas por profesionales.

*Yo, desde mi punto de vista…, yo tenía una depresión*. (Madre 2)

También refieren haber sufrido actos humillantes y vejatorios, de vulneración de los derechos más básicos de madres y criaturas, tanto por acciones directas como por actuaciones en las que no hay una promoción, fomento y apoyo de la lactancia materna, suponiendo una forma de discriminación corporal específica:

*A veces estaban los policías y yo le estaba dando el pecho y me decían: “¡nos vamos!”, y yo decía: “no he terminado”. Y ellos: “que nos tenemos que ir”. Y ellas* [enfermeras] *me decían: “tranquila, nosotras ahora le llenamos un biberón y se lo damos”. Y yo: “vale gracias”. Creo que más que todo son ellos* [los policías], *los que...* (Madre 1)

## CONCLUSIONES

El objetivo principal de este artículo, como señalamos al inicio, es analizar la experiencia con respecto a la lactancia materna de madres privadas de libertad en cárceles del sistema penitenciario español, con interés en la posible percepción de prácticas alusivas a la violencia obstétrica en la etapa perinatal. Así, se pretendía ahondar en la experiencia de la lactancia materna, durante la gestación, el parto y el puerperio de mujeres (y sus criaturas) encarceladas en diferentes Unidades de Madres ubicadas en territorio español, en aras de documentar y comprender con más luz una situación problemática fundamental y, en su caso, poder formular recomendaciones oportunas e informadas.

En función de ello, y tras el recorrido analítico de nuestros principales resultados, encontramos que se necesitan políticas públicas y penitenciarias que se alineen con las sugerencias de la OMS (los bebés deben ser amamantados exclusivamente durante los primeros seis meses de vida, para después introducirse alimentos complementarios nutricionalmente adecuados y seguros, mientras se continúa con lactancia materna hasta los dos años de edad, como mínimo); que dicten la implementación de estrategias que aseguren el éxito de la lactancia materna; y que, además, pongan fin a la grave vulneración de derechos humanos a la que se encuentran expuestas: derecho a la alimentación y la salud física, psicológica y emocional, así como derechos sexuales y reproductivos, ya que la lactancia materna forma parte esencial del ciclo sexual de las mujeres madres.

En este sentido, también debieran generarse e implementarse políticas concretas que eviten todas aquellas prácticas físicas violentas por acción u omisión (actos no informados, no apropiados, no consensuados, no consentidos) y psicológicas (trato paternalista, autoritario, humillante, vejatorio), que hagan alusión a la violencia obstétrica y, por tanto, supongan una violación de derechos humanos.

Es necesaria la formación y la actualización en la materia que nos ocupa de todos los agentes implicados durante el encarcelamiento, para que puedan velar por el bienestar de madres y criaturas durante la etapa perinatal y sean capaces de llevar a cabo acciones de promoción, protección y apoyo a la lactancia materna, de manera efectiva y significativa.

Así mismo, urgen profesionales de la salud mental perinatal en los centros penitenciarios en los que haya mujeres cumpliendo condena durante este periodo, que puedan ofrecer respuesta a las necesidades concretas que se presentan en dicha etapa. Y es que, aunque en esta investigación hayamos encontrado dificultades para las madres encarceladas, análogas en muchos sentidos a las que padecen el resto, el elemento crucial que se ha revelado es que esta problemática se suma -insistiendo en esa mirada interseccional- a un complejo entramado de discriminación, donde la inequidad se agrava de forma singular. 

Los hallazgos de este trabajo, si bien parciales y en elaboración, suponen un valor para la contribución al corpus de conocimientos sobre maternidad en cárceles, sobre el período perinatal (tan complejo y delicado) de madres-criaturas en esta situación, y, al fin, sobre la lactancia misma, ese fenómeno básico para la salud de la especie y derecho fundamental de cualquier criatura, así como de cualquier mujer que se convierte en madre. Por ello, se hace necesario seguir abriendo camino en este campo, con el fin de continuar explorando esta población y profundizando en las diferentes cuestiones que conforman el objetivo de la investigación y que requerirían un mayor detenimiento. Por ejemplo, se habría de profundizar en las vivencias y experiencias en torno a la violencia obstétrica, en general, y el abordaje de la salud mental perinatal, en particular, en el ámbito penitenciario.

Por todo ello, la conclusión fundamental apunta a la necesidad imprescindible de aplicar políticas penitenciaras con perspectiva de género y feminista que, además de estar orientadas a erradicar las graves desigualdades y discriminaciones que sufren las mujeres presas, sirvan para proteger los derechos más básicos de madres y criaturas, ofreciendo una respuesta efectiva a su realidad y acorde con los derechos humanos: también, y muy especialmente, en relación con su práctica lactante, en tanto que es un derecho humano básico.

## References

[1] Almeda E (2017). Criminologías feministas, investigación y cárceles de mujeres en España. Papers.

[2] Massó E (2015). Una etnografía lactivista: la dignidad lactante a través de deseos y políticas. AIBR. Revista de Antropología Iberoamericana.

[3] Ribeiro SG, Lessa PRA, Martings MH, Nicolau AO, Fernandes AFC, Pinheiro AKB (2013). Experiência do amamentar por mães privadas de liberdade: estudo exploratório descritivo. Enfermagem em Foco.

[4] Dalmácio LM, Cruz EJS, Cavalcante LIC (2014). Percepções de mães encarceradas sobre o direito á amamentação no sistema prisional. Revista Brasileira de História & Ciências Sociais.

[5] Cavalcanti A, Cavalcanti G, Celino S, Corrêa R, Ramos R, Cavalcanti A (2018). Born in Chains: Perceptions of Brazilian Mothers Deprived of Freedom about Breastfeeding. Pesquisa Brasileira em Odontopediatria e Clínica Integrada.

[6] Guimarães ML, Guedes TG, Lima LS, Morais SCRV, Javorski M, Linhares FMP (2018). Promotion of breastfeeding in the prison system from the perception of incarcerated nursing mothers. Texto & Contexto - Enfermagem.

[7] Mariano GJS, Silva IA (2018). Significando o amamentar na prisão. Texto & Contexto - Enfermagem.

[8] Santos MV, Alves VH, Rodrigues DP, Vieira BDG, Marchiori GRS, Branco MBLR, Oliveira TR, Bonazzi VCAM (2022). Breastfeeding booklet in prison institutions: initiative for promotion, protection and support. Revista Brasileira de Enfermagem.

[9] Santos MV, Alves VH, Rodrigues DP, Tavares MR, Guerra JVV, Calandrini TSS, Marchiori GRS, Dulfe PAM (2022). Promoção, proteção e apoio ao aleitamento materno no espaço prisional: uma scoping review. Ciência & Saúde Coletiva.

[10] Santos MV, Alves VH, Pereira AV, Vieira BDG, Rodrigues DP, Tavares MR, Calandrini TSS, Ferreira EA (2022). The vital value of breastfeeding for imprisoned women. Texto & Contexto - Enfermagem.

[11] Huang K, Atlas R, Parvez F (2012). The significance of breastfeeding to incarcerated pregnant women: an exploratory study. Birth.

[12] Shlafer RJ, Davis L, Hindt LA, Goshin LS, Gerrity E (2018). Intention and initiation of breastfeeding among women who are incarcerated. Nursing for Women’s Health.

[13] Asiodu IV, Beal L, Sufrin C (2021). Breastfeeding in incarcerated settings in the United States: A National Survey of Frequency and Policies. Breastfeeding Medicine.

[14] Wouk K, Piggott J, Towner Wright S, Palmquist AEL, Knittel A (2022). Lactation support for people who are incarcerated: A systematic review. Breastfeeding Medicine.

[15] Almeda E (2017). El ayer y hoy de las cárceles de mujeres en España. Revista Pena y Estado.

[16] Fochi MCS, Higa R, Camisão AR, Turato ER, Lopes MHBM (2017). Vivências de gestantes em situação de prisão. Revista Eletrônica de Enfermagem.

[17] Paynter MJ, Drake EK, Cassidy C, Snelgrove-Clarke E (2019). Maternal health outcomes for incarcerated women: A scoping review. Journal of Clinical Nursing.

[18] Cross J (2020). Imprisoning pregnant and parenting women: a focus on social justice, equal rights, and equality. Health & Social Work.

[19] Alves IC, Hordones L, Lopes LM (2019). No cadeião não tem nada disso não: a percepção da saúde pelas internas do Centro de Referência à Gestante Privada de Liberdade. Revista Brasileira de Ciências Criminais.

[20] Friedman SH, Kaempf A, Kauffman S (2020). The realities of pregnancy and mothering while incarcerated. The Journal of the American Academy of Psychiatry and the Law.

[21] Almeda E, Bodelón E (2007). Mujeres y castigo un enfoque socio-jurídico y de género.

[22] United Nations (2016). Joint statement by the UN Special Rapporteurs on the Right to Food, Right to Health, the Working Group on Discrimination against Women in law and in practice, and the Committee on the Rights of the Child in support of increased efforts to promote, support and protect breast-feeding.

[23] Massó E (2021). Human lactation in the anthropocene: a post-pandemic emergency. Medica Review.

[24] Guimarães ML, Guedes TG, Lima LS, Morais SCRV, Javorski M, Linhares FMP (2018). Promoção da aleitamento materno no sistema prisional a partir da percepção de nutrizes encarceradas. Texto & Contexto - Enfermagem.

[25] Organización Mundial de la Salud (2001). Estrategia mundial para la alimentación del lactante y del niño pequeño. 54ª Asamblea Mundial de la Salud.

[26] Massó E (2015). Conjeturas (¿y refutaciones?) sobre amamantamiento: Teta decolonial. Dilemata.

[27] León-Cava N, Lutter C, Ross J, Martín L (2022). Cuantificación de los beneficios de la lactancia materna: Reseña de la evidencia.

[28] Organización Mundial de la Salud (2014). Prevención y erradicación de la falta de respeto y el maltrato durante la atención del parto en centros de salud.

[29] Sadler M, Quattrocchi P, Magnone N (2020). Violencia obstétrica en América Latina: conceptualización, experiencias, medición y estrategias.

[30] Naciones Unidas (2022). España responsable por violencia obstétrica, según el Comité de derechos de las mujeres de la ONU.

[31] Quattrocchi P, Quattrocchi P, Magnone N (2020). Violencia obstétrica en América Latina: conceptualización, experiencias, medición y estrategias.

[32] Igareda N, Bodelón E, Nicolás G (2006). Género y dominación: Críticas feministas del derecho y el poder.

[33] Crenshaw K (1989). Demarginalizing the intersection of race and sex: A black feminist critique of antidiscrimination doctrine, feminist theory and antiracist politics. University of Chicago Legal Forum.

[34] Baldwin A, Sobolewska A, Capper T (2018). Pregnant in prison: An integrative literature review. Women and Birth.

[35] Bülow W, Lindblom L (2020). The social injustice of parental imprisonment. Moral Philosophy and Politics.

[36] Foucault M (1979). Vigilar y castigar.

[37] Abejón S (2021). Males fembres pecadores: Genealogia de la història del càstig i de les presons de dones a Barcelona.

[38] Ministerio del Interior de España (2021). Anuario Estadístico.

[39] España (1979). Ley Orgánica 1/1979, de 26 de septiembre, General Penitenciaria.

[40] Asociación Proderechos Humanos de Andalucía (2021). Informe sobre la situación de las mujeres presas.

[41] Martínez R, Aguilera M (2018). XX Jornadas de los Servicios de Orientación y Asistencia Jurídica Penitenciaria (SOAJP).

[42] Valdez Z (2013). Etnografía crítica. Surgimiento y repercusiones. Revista Comunicación.

[43] Ibarra-Sáiz MS, González-Elorza A, Rodríguez-Gómez G (2023). Aportaciones metodológicas para el uso de la entrevista semiestructurada en la investigación educativa a partir de un estudio de caso múltiple. Revista de Investigación Educativa.

[44] Taylor SJ, Bogdan R (1987). Introducción a los métodos cualitativos de investigación: La búsqueda de significados.

[45] Glaser BG, Strauss A (1967). The discovery of grounded theory: Strategies for qualitative research.

[46] Osses S, Sánchez I, Ibáñez FM (2006). Investigación cualitativa en educación: hacia la generación de teoría a través del proceso analítico. Estudios Pedagógicos (Valdivia).

[47] Viveros M (2016). La interseccionalidad: una aproximación situada a la dominación. Debate Feminista.

[48] García D, Ruiz M (2021). Un viaje por las emociones en procesos de investigación feminista. Empiria: Revista De metodología De Ciencias Sociales.

[49] España (1995). Ley Orgánica 10 de 1995 del Código Penal.

[50] Yagüe Olmos C (2007). Mujeres en prisión: Intervención basada en sus características, necesidades y demandas. Revista Española de Investigación Criminológica.

[51] Bronfenbrenner U (1987). La ecología del desarrollo humano.

[52] Ainsworth MDS (1969). Object relations, dependency, and attachment: A theoretical review of the infant-mother relationship. Child Development.

[53] Bowlby J (1969). Attachment and loss.

[54] Valverde J (1997). La cárcel y sus consecuencias: La intervención sobre la conducta desadaptada.

[55] Olza I, Ruiz-Berdún D, Villarmea S (2017). La culpa de las madres: Promover la lactancia materna sin presionar a las mujeres. Dilemata.

[56] Olza I, Serrano E, Muñoz C (2011). Lactancia para psiquiatras: Recomendaciones sobre el empleo de psicofármacos en madres lactantes. Archivos de Psiquiatría.

[57] Fernández IM, Fernández CT (2013). Lactancia materna: prevención de problemas tempranos en las mamas mediante una técnica de amamantamiento eficaz. Enfermería Global.

[58] Morgan R (1970). Sisterhood is powerful: An anthology of writings from the women’s liberation movement.

[59] Massó E (2018). “Le salvé la vida”: el pecho vivido, la leche narrada. Historia(s) de ama de teta, sur de España, siglo XX. Mana.

[60] López de Aberasturi A, Santos N, Ramos Y, García M, Artola C, Arara I (2021). Prevalencia y determinantes de la lactancia materna: estudio Zorrotzaurre. Nutrición Hospitalaria.

[61] Massó E (2013). Lactancia materna y revolución, o la teta como insumisión biocultural: calostro, cuerpo y cuidado. Dilemata.

[62] Martín-Ramos S, Domínguez-Aurrecoechea B, García Vera C, Lorente García Mauriño AM, Sánchez Almeida E, Solís-Sánchez G (2024). Lactancia materna en España y factores relacionados con su instauración y mantenimiento: estudio LAyDI (PAPenRed). Atención Primaria.

[63] López EP (2022). Estudio bibliométrico sobre mitos y verdades de la lactancia materna. Npunto.

[64] Calama Valero JM, Aguayo Maldonado J (2009). Manual de lactancia materna.

[65] Ausona M (2013). “Encara li dones el pit?” Tabús occidentals envers els usos de la corporalitat en la criança. Quaderns-E.

[66] Olza I, Fernández P, González A, Herrero F, Carmona S, Gil A, Amado E, Dip ME (2021). Propuesta de un modelo ecosistémico para la atención integral a la salud mental perinatal. Revista de la Asociación Española de Neuropsiquiatría.

[67] Antúnez M, Martín N, Casilari JC, Mérida FJ (2022). Depresión posparto, análisis de los factores de riesgo y la intervención de Enfermería: Revisión bibliográfica. Enfermería Cuidándote.

[68] O’Hara MW (1986). Social support, life events, and depression during pregnancy and the puerperium. Archive of General Psychiatry.

