# Supplementary material for: Lactancia materna entre rejas: experiencias de las madres encarceladas en el sistema penitenciario español
Source: Salud Colect. 2024 Feb 29;20:e4665. [Article in Spanish] doi: 10.18294/sc.2024.4665 (PMC11822911; doi:10.18294/sc.2024.4665)
Supplement: Translation

# Translation: Breastfeeding behind bars: Experiences of incarcerated mothers in the Spanish penitentiary system

**Authors:** Pilar Jubany-Roig, Ester Massó Guijarro

**Affiliations:** 1 Psychologist. Doctoral Researcher, Doctoral Program in Philosophy, Universidad de Granada, Spain. proigjubany@correo.ugr.es; 2 Doctora en Filosofía y Antropología. Profesora titular de Filosofía Moral, Universidad de Granada, España. ester@ugr.es

**Keywords:** Prisons, Breastfeeding, Obstetric Violence, Human Rights, Feminism, Intersectionality, Mental Health, Spain

## Abstract

This research aims to analyze the breastfeeding experiences of incarcerated mothers in the prisons of the Spanish penitentiary system. Additionally, it explores whether these mothers have perceived practices related to obstetric violence during pregnancy, childbirth, and the postpartum period. An exploratory-descriptive study was conducted using a qualitative approach and a critical ethnographic method. Fieldwork, including participant observation and semi-structured interviews, was carried out between December 2021 and April 2022. The study involved 30 adult women from Africa, Europe, Eastern Europe, and Latin America, all serving sentences with their infants in Mother Units located in the Spanish cities of Alicante, Barcelona, Madrid, and Seville. The main findings highlight the need for penitentiary policies with a gender and feminist perspective. These policies should aim to eliminate severe inequalities and discriminations faced by incarcerated women while protecting the basic rights of both mothers and infants.

## INTRODUCTION

Neither women’s prisons nor the situation of incarcerated women in Spain were studied in depth until the late 1980s.^1^^)^ Until then, these issues were largely overlooked, resulting in a significant gap in the literature.

The issue of breastfeeding, however, has garnered interest from diverse perspectives, leading to the development of significant bodies of knowledge on motherhood and child-rearing.^2^ Nevertheless, few studies have explored the connections between these issues. That is, little research exists on breastfeeding in the Spanish prison system, in contrast to cases like Brazil^3^^,^^4^^,^^5^^,^^6^^,^^7^^,^^8^^,^^9^^,^^10^ and the United States,^11^^,^^12^^,^^13^^,^^14^ for instance. 

Therefore, we have identified a critical gap in the literature regarding the views and experiences related to breastfeeding on the part of incarcerated women in Spain, with a particular emphasis on situations of obstetric violence in general. Such situations may negatively affect these women by interfering with or hindering breastfeeding, despite being recognized as a human right.

As presented in this article, our research on breastfeeding among incarcerated women constitutes - both methodologically and phenomenologically - the first instance of mothers being interviewed about breastfeeding within prison walls. More specifically, employing a qualitative and feminist approach, we have aimed to capture first hand their unique voices, narratives, and worries, and explore their possible traumatic experiences of obstetric violence in the first person.

History shows that the internal structures and dynamics of women’s prisons, as described by Almeda:

have had, and continue to have, their own history, philosophy, operational logic, and features, because over the centuries there have been differing approaches to punishing men and women who have violated criminal laws.^15^

The incarceration of pregnant and postpartum women has been examined in numerous studies, revealing severe deficiencies in maternal and perinatal care and the inadequacy of facilities.^16^^,^^17^^,^^18^^,^^19^^,^^20^ In fact, both prisons and the studies about them are often based on assumptions that are both gender-neutral and universal, which in reality are tacitly and implicitly based on male experience. Therefore, prisons continue to

adopt falsely masculine, universal patterns, continuing to discriminate against and penalize women for being women […]. Through a mechanism of social domination that, alongside others, perpetuates the social subordination of all of us as women.^21^

In 2016, the United Nations Human Rights Council^22^ recognized breastfeeding as a *human right* both for mothers and children that should be promoted and supported in all circumstances. Thus, there is a need to comprehend it based on a strong sense of *universality* with clear feminist connotations.^23^ The violation of such a basic right should *not occur even when* mothers and their offspring are incarcerated,^24^ as the best interests of the child must always be prioritized. 

Breastfeeding is therefore a matter of human rights, given that it

is an unequalled way of providing ideal food for the healthy growth and development of infants; it is also an integral part of the reproductive process with important implications for the health of mothers.^25^

Breastfeeding is an integral part of the essential care for newborn human beings - at least during the first few months of life - and the primary mode of nutrition up to age two according to the World Health Organization (WHO). It therefore involves a bio-cultural phenomenology^26^^)^ which transcends the simple act of feeding and nutrition, touching the very core of human health.^23^

According to international organizations and established scientific evidence, it is possible to confirm the benefits of breastfeeding at a global level.^27^^)^ Its numerous physical and emotional benefits for children and mothers both in the short- and long-term are not questioned. As such, rather than emphasize the *benefits* of breastfeeding, we can point out the *risks* of not doing so at a planetary scale in the Anthropocene, given that this practice represents the physiological form of human development during exterogestation. This is so to the point that it is possible to quantify these risks and advantages with the *Cost of Not Breastfeeding Tool*,^23^^)^ which aims to provide policymakers and advocates with a tool for estimating the potential health, human capital, and economic benefits of expanding breastfeeding promotion and support strategies. Milk production after childbirth is, in fact, a physiological process (lactogenesis begins during pregnancy), considered a part of mothers’ sex-reproductive lives, and is greatly influenced by socio-cultural and anthropological factors.^23^

On the other hand, obstetric violence has been recognized by the WHO^28^ as:

a specific form of violence from health professionals (mainly doctors and nursing staff) towards pregnant women, during childbirth or puerperium. It is a violation of women’s reproductive and sexual rights.^28^^)^

Sadler^29^ observes that “although it has not been widely recognized by medical associations, it is widely utilized by women’s organizations seeking to put it on the public agenda.” Additionally, there is increasing evidence that it is a real and verifiable phenomenon: the United Nations^30^ has held Spain responsible for this type of violence against a number of mothers, which has been defined as:

…a violation of human rights, the right to health, and reproductive rights. It is related to the respect for the processes of childbirth, women’s bodies, timeframes, privacy, integrity, autonomy and freedom of choice, as well as all of their rights.^31^

Taking these considerations into account, the main objective of the research that gives rise to this article was to study the breastfeeding experience of mothers incarcerated in the Spanish prison system, with an emphasis on the potential perceptions of practices related to obstetric violence during pregnancy, childbirth, and postpartum, and associated with difficulties in breastfeeding practice.

The motivation driving this research is to contribute to the body of knowledge on this issue for its practical application to improve public policies concerning maternal and infant health among the incarcerated population. From an intersectional perspective,^32^^)^ we argue for the need to address these issues in prison environments, as both the typical challenges of breastfeeding and the potential occurrences of obstetric violence are likely exacerbated in an environment already highly prone to various forms of discrimination.

### Conceptual-epistemological framework: prisons, women, and mothers, in intersectional discrimination

Despite the existence of penitentiary law that contemplates the possibility that incarcerated women

…may be pregnant or have children who are breastfeeding, there are few regulations in comparison with the number of articles regarding other aspects of life in prison (for example, prison labor, the organization and management of commissaries, etc.).^33^

In a similar vein, Baldwin^34^ argues that there is a limited understanding of mothers’ needs on the part of correctional institutions and providers of motherhood services, as well as a general lack of attention to these needs. 

On the other hand, recent research has reflected on the well-being of children incarcerated alongside their mothers, examining whether the inequalities of childhood experiences marked by incarceration are justifiable from a social justice perspective.^35^ Therefore, maternal and child health policies promoting qualified and safe healthcare are of vital importance, especially with regards to reproductive rights, such as support for nutrition, childcare, and above all breastfeeding.^9^^,^^10^

If we examine the history of prisons themselves, existing literature portrays them as relatively recent punitive structures, as it wasn’t until the late 18^th^ century that institutions emerged to subject convicted individuals to a regime of isolation and deprivation of liberty;^36^ although it is worth noting that a school of historiography exists which locates the roots of imprisonment practices in late-13^th^ century Christian orthodoxy.^37^

According to data from the latest Statistical Yearbook^38^ published by Spain’s Ministry of the Interior, by the end of 2021 there were 55,097 people incarcerated in the country’s prisons. Of these, 92.9% were men and 7.1% were women. Of the approximately 80 state-run prisons, only four were women’s prisons (Wad-Ras in Barcelona, Alcalá de Guadaíra in Seville, Madrid-I in Madrid, and Brieva in Ávila). This implies that, despite the fact that Organic Law 1/1979^39^ establishes *exclusive* prisons for women, in practice female prisoners must serve out their sentences in female modules within men’s prisons.

History shows that the configuration and internal dynamics of women’s correctional institutions

…have had, and continue to have, their own history, philosophy, operational logic, and features, because over the centuries there have been differing approaches to punishing men and women who have violated criminal laws.^15^

In this way, women are subjected to multiple punishments: personal, being distanced from their families and for abandoning their traditionally assigned roles as “caring mother/daughter/wife;” social, for having transgressed socially expected norms for women; and that which is related to the crime itself, often having to endure harsher conditions than men during their sentence. All of this is further aggravated in the case of mothers, according to a 2021 report by the Human Rights Association of Andalucía.^40^

Aguilera and Martinez^41^^)^ observe that 80% of imprisoned women are mothers. Prior to the reform of Spain’s General Penitentiary Organic Law in 1996, mothers were allowed to remain in prison with their children until they reached the age of six. However, after this change in the legislation, children were only allowed to remain in correctional facilities until age three. Only in a minority of these cases - and only when the child’s age and the mother’s penal situation permits it - do mothers live with their children behind bars, in so-called Mother Units. Different types of Mother Units exist: internal (pavilions within prisons), external (buildings annexed to the prisons), mixed (blocks where both parents serve their sentences), and dependent (centers for mothers under a regime of semi-liberty) (see Table 1).

**Table 1:** Number of imprisoned mothers and children, according to type of Mother Unit in the Spanish penitentiary system. 2022.

Administrator	Type of Mother Unit	Location	Number of mothers (n=79)	Number of children (n=82)
General Secretary of Penitentiary Institutions, Ministry of the Interior	Internal	Madrid VI (Aranjuez)	24	24
	External	Mallorca	1	1
	External	Madrid	5	5
	External	Sevilla	10	11
	External	Alicante	16	16
	Mixed	Madrid VI (Aranjuez)	12	12
	Dependent	Madrid VI (Aranjuez)	4	5
Department of Justice (Catalonia)	Internal	Barcelona (Wad-Ras Prison)	7	8
Department of Equality, Justice and Social Policy (Basque Country)	Internal	Donostia (Martutene Prison)	0	0

Source: Own elaboration.

## METHODS

An exploratory-descriptive study with a qualitative approach was carried out in four institutions located in four different areas of Spain: External Mother Units (UME) in Alicante, Madrid, and Seville, and the Mothers’ Division within the Wad-Ras Prison in Barcelona. Fieldwork was conducted between December 2021 and April 2022. 

Since 1983, the Spanish government has had a Central Prison Administration for all penitentiary institutions within its territory, with the exception of Catalonia, where the Department of Justice has jurisdiction over penitentiary matters. Similarly, Basque Country took control of administering its prisons in May 2021. 

The study population was made up of 30 adult women who were serving their prison sentences in different institutions alongside their children. Initial contact was made with the administrators of each Mother Unit, who were provided with information on the study by the researcher responsible for carrying out fieldwork and were asked to voluntarily participate. A total of 30 mothers agreed to participate in the study. The same method was used to establish dates and times that fieldwork would be carried out, and authorization was obtained from the General Secretary of Penitentiary Institutions and Catalonia’s Department of Justice. As required, written informed consent was obtained from all participants who were interviewed for the study. From the early stages of this research project, the design of the work plan was supervised and approved in accordance with the legal framework of the Doctoral Program at the University of Granada, including the revision and approval of ethical aspects.

*Critical* ethnography was employed, which

…has fundamental differences with traditional ethnography and is highly relevant due to the acute social and political critiques it puts forward, and therefore has implications in terms of improvements for the communities under study.^42^

This is consistent with the topic and type of research conducted, related to complex situations of social vulnerability and with a population (interviewees) facing intersectional discrimination.

Primary research techniques included semi-structured interviews^43^ and participant observation.^44^ Semi-structured interviews offer greater flexibility than structured interviews or closed questionnaires, allowing for adjustments in the predefined interview guide to be made by researchers, which was slightly modified as data collection progressed. This was to ensure the necessary adaptability in the qualitative research process, particularly in a challenging environment such as a correctional institution. Lastly, participant observation was conducted in a structured and pragmatic fashion, in order to obtain information on the day-to-day activities and interactions of mothers in their habitual environments.

All interviews were held in a private area in order to ensure confidentiality, and they had a duration of 15 to 70 minutes, with an average of 31 minutes. Interviews were held until reaching the point of saturation; they were recorded in a digital format and then transcribed verbatim. Material was analyzed according to the principles of grounded theory^45^ utilizing MAXQDA software, and the *constant comparison method* was employed, which seeks to “base concepts on data […] and therefore necessitates the critical thinking and creativity of researchers.”^46^ The following emergent categories resulted from this process: 1) the motivation to breastfeed and the perception of breastfeeding agency; 2) difficulties and interferences perceived during breastfeeding, and/or in relation to its support and promotion in the healthcare and prison systems; 3) breastfeeding acculturation and misconceptions about breastfeeding; and 4) situations of obstetric violence.

Due to the fact that this research was conceived from a feminist and gender-based perspective, an intersectional^32^ theoretical framework was constructed, being “the most widespread feminist trope for speaking about either identity or multiple and interdependent inequalities.”^47^ Furthermore, this includes positions that take into account the epistemology of emotions in the research process.^48^

## RESULTS AND DISCUSSION

In this section we present and discuss the results of our research, given that both the points of contact with existing literature on this issue and intersectional and feminist perspectives connect with the results of the study in dynamic and systematic ways.

### Socio-demographic data: age, origin, education/work

The study population (Table 2) was made up of 30 adult women who were serving prison sentences with their children in different correctional centers. Their ages ranged from 19 to 45 years old. Of the women in Mother Units, 53% were in the 31-40 age group, followed by the 18-30 group. In other words, the majority of women were between 18 and 40 years old, which can be generally considered reproductive age. 

**Table 2:** Socio-demographic data of interviewed mothers (n=30), by Mother Units in the Spanish penitentiary system. December 2021 - April 2022.

Socio-demographic data	UME Madrid (n= 4)	UME Alicante (n= 14)	UME Sevilla (n= 6)	DM de Barcelona (n= 6)
Age of mothers				
18 to 30	4	3	1	2
31 to 40	-	8	4	4
41 to 50	-	2	1	-
NR	-	1	-	-
Country of origin				
Algeria	-	1	-	-
Bolivia	-	1	-	-
Brazil	-	1	-	-
Chile	-	-	-	1
Colombia	-	1	-	-
Costa Rica	-	-	-	1
Spain	1	7	5	3
Italy	-	1	-	-
Nigeria	-	1	-	-
Peru	2	-	1	1
Russia	-	1	-	-
Venezuela	1	-	-	-
Educational level				
Primary	-	10	5	3
Secondary	4	2	-	2
University	-	1	1	-
NR	-	1	-	1
Occupation prior to incarceration				
Accountants, administration	2	-	-	-
Business owners	-	2	-	-
Students	-	-	-	-
Geriatric nurses	-	-	1	1
Cleaning personnel	1	2	2	1
Hotel services and salespersons	1	4	1	2
Unemployed	-	6	2	-
NR	-	-	-	2

Source: Own elaboration. UME= External Mother Unit. DM= Department of Mothers. NR= No response.

The mothers interviewed came from Africa, Europe, Latin America, and Eastern Europe; 53% were of Spanish origin. This may be due to the fact that in Mother Units, most women serving sentences are granted some flexibility in penitentiary classification in accordance with Article 100.2 of the Penitentiary Regulation. In some cases they are even granted third-degree classification, meaning that they serve a custodial sentence but under a regime of semi-liberty (except in the Alicante Mother Unit, which houses pre-trial detainees, long-term convicts, those convicted of serious or very serious crimes, or those exhibiting certain maladaptive behaviors or significant mental disorders). Therefore, alternative measures may have been adopted before they were able to serve their sentence in Mother Units, such as deportation in the case of foreign women, according to criteria outlined in Organic Law 10/1995, of 23 November, of the Criminal Code (Article 89).^49^

Regarding educational level, the majority had less than a primary school education (18 of the women), followed by secondary school (8) and university (2). In terms of occupation, 33% of the mothers worked in the hotel sector, 20% were homemakers, and 16% were cleaning personnel. According to Yagüe,^50^^)^ analyses of the socioeconomic background of incarcerated women show that they come from the most disadvantaged sectors of society, with a clear history of discrimination and exclusion behind them: fractured families (on the brink of poverty), issues with drug abuse, limited or nonexistent education and professionalization, victims of gender-based violence, heavy burdens of family responsibility, among others. These circumstances, in addition to putting women at risk for engaging in criminal behavior, impede potential reinsertion after they have served their sentences, plunging them into a cycle of poverty and marginalization. In short, prison “excludes women who were not excluded prior to incarceration and exacerbates the marginalization of those who were already marginalized.”^33^

The data in Table 3 show that a total of 30 women were serving a prison sentence alongside a son or daughter, and one of them was pregnant; a few mothers had two children in prison. It is important to note that in addition to being incarcerated with one or more children, these women are also responsible for children outside of the facilities: 43% had more than two children outside of prison, followed by 20% with one or two children, and 13% with no children outside of prison. The lack of Mother Units results in a dispersion that leads to greater difficulties in maintaining contact with their children not in prison, and “inevitably, women have to endure the loss of emotional ties due to the separation from their sons and daughters.”^33^

**Table 3:** Pregnancy status, number of children in prison and their ages, and number of children outside of prison of interviewed mothers (n=30), by Mother Units in the Spanish penitentiary system. December 2021 - April 2022.

	UME Madrid (n= 4)	UME Alicante (n= 14)	UME Sevilla (n= 6)	DM de Barcelona (n= 6)
Pregnancy status				
Has children, not pregnant	4	14	6	6
Has children, is pregnant	-	1	-	-
Children in prison				
1 child	4	14	5	5
2 children	-	-	1	1
Age of children in prison (months)*				
0 to 6	-	4	1	2
7 to 12	2	1	2	1
13 to 24	1	5	1	1
25 to 36	1	3	1	2
37 and over	-	-	1	-
NR	-	1	-	1
Children outside of prison				
0 children	2	2	-	-
1 child	1	1	1	2
2 children	1	4	1	1
Over 2 children	-	7	4	2
NR	-	-	-	1

Source: Own elaboration. *The number of children in prison with their mothers is indicated, not the number of mothers who have children in each age group, causing the total to be higher than the number of mothers. UME= External Mother Unit. DM= Department of Mothers. NR= No response.

### Specific data regarding breastfeeding

Regarding breastfeeding, Table 4 shows that 77% of the women interviewed reported having breastfed their child while in prison. Of the 23 women who did, over 50% opted for exclusive breastfeeding while the remainder chose mixed feeding. Regarding the duration, nine of the women breastfed for 0 to 3 months, five did so for 4 to 6 months, none of the mothers did so for the range of 7 to 24 months, and only one did for longer than 24 months. The remainder of women who participated were still nursing at the time of the interview.

**Table 4:** Number of mothers who breastfed, type and duration, by Mother Units in the Spanish penitentiary system. December 2021 - April 2022.

	UME Madrid (n= 4)	UME Alicante (n= 14)	UME Sevilla (n= 6)	DM de Barcelona (n= 6)
Breastfed children in prison				
Yes	3	10	6	4
No	1	4	-	1
NR	-	-	-	1
Type of breastfeeding				
Exclusive	1	6	2	4
Mixed	2	4	4	-
Duration (in months)				
0 to 3	2	4	1	2
4 to 6	-	2	2	1
7 to 12	-	-	-	-
13 to 24	-	-	-	-
25 and over	-	1	-	-
Still breastfeeding	1	3	3	1

Source: Own elaboration. UME= External Mother Unit. DM= Department of Mothers. NR= No response.

As is customary in anthropological-ethnographic studies, we will now present our findings alongside the terms, expressions, and narratives of the women themselves, presenting representative or significant excerpts from interviews verbatim, always preserving the original language and expressions. 

### Emergent categories

#### Motivation for breastfeeding and perceptions surrounding their breastfeeding agency

Consistent with what we presented in the first section of the article, the majority of mothers recognize and value breastfeeding their children, but different viewpoints are evident in their narratives, related to internal or external motivations. 

Some of them expressed a desire to feel first-hand what breastfeeding was like and the experience of skin-to-skin contact:

*I love it. I wasn’t going to nurse him because it had been so long* [without breastfeeding], *my daughter for example is much bigger. But I just love being there with them.* (Mother 10)

Others considered breastfeeding to be the best form of nutrition for their children to grow in a healthy way:

*Unconsciously, since I am a mother, I know that breastfeeding is the best thing there is for the baby’s stomach, and so I always nursed my kids.* (Mother 3).

They also alluded to breastfeeding as a way to establish and maintain a mother-child bond:

*To me it’s so beautiful. I think… I respect everyone and each person should do what they want, but I think children… need it. It’s a good thing… it’s a bond that you create with your child. And, I mean, she loves it.* (Mother 2)

This last point is particularly important in a context where there is a high likelihood of separation and the fear that this can happen is always present. Therefore, breastfeeding can contribute to generating what Bronfenbrenner^51^ calls a healthy ecosystem that encourages healthy attachment.^52^^,^^53^

Moreover, breastfeeding is often experienced as a form of empowerment, as it implies reclaiming “agency over one’s own body.”^26^ Something similar goes on within prison walls, where breastfeeding proves to have a positive effect on incarcerated women, given the systematic removal of their decision-making power in this context (“*they even decide when I get to call my family*”). It provides them with a chance to freely occupy a space (“*I can breastfeed anywhere in the prison*”), since prison is a space in which limits and prohibitions are constantly imposed upon them. This could imply a potentially protective aspect, as prison constitutes a limitation both on on the lives of prisoners as well as their bodies, with severe physical, mental, emotional, and social consequences.^54^

Several mothers expressed a desire to have been able to experience this, although on some occasions they were not able to fulfill this desire:

*I would have liked to do it for more time. Because I see mothers nursing their baby and the truth is it makes me wish for that. But it wasn’t meant to be for me.* (Mother 23).

Moreover, not being able to breastfeed their children caused intense feelings of sadness for them:

*Man, it was a real shame. I cried my eyes out when they told me that my milk wasn’t enough and my baby wasn’t gaining weight. Because I just wanted to breastfeed. I wanted to try it and I couldn’t.* (Mother 20).

They even allude to the fact that this was a source of trauma for them:

*I say: you should have done it, you should have continued. Here there are girls who nurse with no problem at all, they are used to prison, but I’m not. To me it’s traumatic* [not having been able to nurse]. (Mother 9).

Feelings of guilt regarding not breastfeeding their children were also evident, which can negatively impact their relationship with their children and/or their self-esteem. This is an issue that has been dealt with for some time within the critical promotion of breastfeeding.^55^

*When I got here and tried it, it worked, it was my fault because I didn’t try hard enough. I feel bad.* […] *my daughter had a hard time feeding. Her weight was good, around average and everything, but it was hard for me because I raised them really well that way. Later on I would give them their little mash, the pastas we make… and to see that I couldn’t do that with my daughter hurts, it hurts a lot.* (Mother 9).

Furthermore, they expressed knowledge about the fact that in the act of breastfeeding a strong bond is created and that is very beneficial in terms of cultivating healthy mother-child attachment.^52^^,^^53^

*To me it’s so beautiful. I think… I respect everyone and each person should do what they want, but I think children… need it. It’s a good thing… it’s a bond that you create with your child. And, I mean, she loves it.* (Mother 2)

#### Difficulties and interferences perceived during breastfeeding, and/or in relation to its promotion and support in the healthcare and penitentiary systems

It is well known that in the majority of cases where breastfeeding is not successfully carried out, this is not directly caused by the behavior or actions of mothers, but rather by different interferences and a lack of adequate support in their immediate surroundings. This is particularly relevant in the case of incarcerated women, who already face extreme vulnerability and are in much more disadvantageous conditions in comparison to mothers who experience this while free.

Some of the difficulties that they express/experience have to do with the possibility that they could be separated from their infant at some point in time; for example, due to detainment:

*I didn’t breastfeed because when I left my daughter’s side she was a month old and then they brought her to me at three months*. (Mother 13)

Another barrier that mothers can encounter during this time is that medical processional recommend they stop breastfeeding under the pretense of administering certain medications, similar to what happens outside of prisons. 

On occasion, these recommendations are not based on up-to-date scientific evidence, and “unfamiliarity with the benefits of breastfeeding and with recent studies on pharmacokinetics in breast milk often leads to psychiatrists or primary care physicians to recommend early weaning so that a mother can begin psychopharmacological treatment.”^56^^)^

*I wanted to continue. But look, it was more important to get* [the placenta] *out and make sure nothing stayed inside me. I haven’t stopped since yesterday when they told me… it’s just that I was expecting good news, like look, you don’t have it anymore. And what they told me scared me. That they might have to do surgery, maybe open me up again. It sucks.* […] *You can’t anymore. That’s what they told me. And I said: I have to stop nursing? Yes.* (Mother 5)

The mental health of mothers is compromised due to multiple factors which are far more common among incarcerated individuals, who are more vulnerable to them: “pregnancies are often unplanned and there are complications due to lack of prenatal care, maternal trauma, poor nutrition, substance abuse, mental disorders, chronic medical conditions, low socioeconomic status, and limited social support.”^20^ The combination of all these factors frequently leads these mothers to require psychopharmacological treatment. 

There is solid evidence that some antidepressants, anxiolytics, and even lithium carbonate can be compatible with breastfeeding when used with caution,^56^ as demonstrated by the *e-lactancia* project led by the Association for the Promotion of and Scientific and Cultural Research into Breastfeeding (APILAM). 

However, there are still healthcare professionals that advise against breastfeeding without truly well-founded arguments, violating the premise that “the benefits of breastfeeding far exceed those of artificial feeding, so healthcare professionals should be aware of these benefits and promote the continuity of breastfeeding whenever possible.”^56^

Regarding the mental health of mothers, it should be noted that one of the Mother Units has a perinatal mental health project, a program that addresses mental disorders experienced by women during pregnancy and/or in the first 12 months postpartum. However, we found that these interventions were not sufficient or timely for the target population, given that the mothers in this Unit did not *identify* the interventions conducted and expressed having felt a lack of psychological support during the perinatal stage.

It is likely that the extremely difficult circumstances of being deprived of their freedom, along with the hostile environment they face in prison, lead to specific interferences with exclusive breastfeeding, since this is a complex and fragile process which *necessitates* that the mother *feel* she is able to do so, as well as have the desire to do so. In many cases, learned defenselessness is evident:

*I couldn’t with my daughter, I’m going to be honest with you, first because I didn’t feel alright. I wasn’t ok psychologically. I had to accept something that was very difficult. The atmosphere here… all of that because this is still a prison, after all. You have to always have your armor. If you have to worry about so many things and on top of that always be ready to fight, with all of that… I didn’t feel ready for it.* (Mother 9).

On the other hand, some of the mothers report having experienced serious difficulties due to physical pain while breastfeeding their infants, due to improper latch, ineffective positioning, and so on. Evidence shows that there is an association between early problems and poor technique, with “correct breastfeeding technique forming the basis of successful breastfeeding and prevention of breastfeeding-associated breast problems such as nipple fissures, breast engorgement, or mastitis.”^57^

*It’s just that the pain that I felt during the two critical weeks with the bleeding, the swelling… Look, they even gave me these things over at the hospital* [nipple shields]. *Because I felt like I was dying.* (Mother 5)

Regarding the perception of having received help when facing potential difficulties during their experience, the narratives suggest that in most cases there is not a strong sense of having received effective support from staff (healthcare or otherwise) in Mother Units. Despite the critical importance of this assistance in breastfeeding practice, there are not always strategies for promotion, protection, and support in prisons - or outside of prison, for that matter - which successfully reach incarcerated women in a meaningful and effective way.^9^ This could largely explain the worldwide social hypogalactia that has been analyzed and described, stemming from widespread social acculturation surrounding breastfeeding:^23^

*I really wanted to nurse, but it was difficult because I didn’t have anyone to help me.* (Mother 14)

*Nobody. I couldn’t talk to anybody. It was all just ‘bottle, drink the milk’ and that’s it. Problem solved.* (Mother 22)

It was also possible to detect a significant lack of support when mothers were hospitalized after giving birth:

*I would have loved it if someone said to me, “don’t worry, we’ll try using a pump. We are going to start over here and you’ll see that it’s all going to be ok.” Besides, F. was so tiny when he was born, I was so scared.* (Mother 22)

Several mothers reported asking for help from healthcare professionals, both within and outside the prison, and they recommended using formula instead of breast milk:

*Here they give you the milk and the bottle, they give you everything. Because the doctor* [at the Mother Unit] *told me that she was always still hungry.* (Mother 8)

Nevertheless, in some narratives the women mentioned on occasion encountering healthcare professionals that encouraged breastfeeding and supported them when they had difficulties, either staff at the detention center or external midwives:

*They would come here to give talks. About 10 or 15 days ago they came to give a talk, a midwife, the same one that takes care of us, about what are contraceptives, about breastfeeding and all that.* (Mother 29)

In their “*herstories*”^58^ - a feminist notion derived from a play on words literally meaning “*her* story,” which has been employed by Massó to reconstruct life histories of breastfeeding mothers/“lactivists” - it is clear that these mothers turn to friends and acquaintances (peers) when they have questions or problems during breastfeeding, even before consulting with a medical professional. This is due to the fact that they trust in the experience of *others* (their peers, their equals) who are already mothers, because they feel more comfortable sharing their uncertainties with a woman similar to them. This is analogous to the logic of *lactivism* (pro-breastfeeding activism), which “displays a unique combination of the traditionally dualistic arenas of the public-private or nature-culture, constituting a transformative politics of bodies, customs, and societies:”^2^

*We ask each other questions. I say to them: “put on some lotion or something.” Yeah, here we help each other out.* (Mother 12)

In fact, there is explicit discourse regarding the gender of healthcare professionals and which are their most trustworthy sources of information:

*There was a pediatrician, but that’s for the kids and not for us. And when you had a question, they would say, look: “the doctor comes every other Thursday.” But it just didn’t feel as normal, talking to a female doctor, as to a male one. I didn’t feel the same. So I decided not to talk.* (Mother 10)

All of this shows that even today there is a lack of (or not enough) healthcare personnel with up-to-date training on breastfeeding, despite numerous studies (biological and epidemiological) showing that the decision to not breastfeed has significant negative effects on the nutrition, development, and health of both the baby and the mother (as we pointed out at the beginning). This signifies that breastfeeding is the healthcare intervention that obtains the largest health benefit for the lowest economic cost;^60^^)^ or, in other words, it is the healthcare practice with the most advantageous cost-benefit ratio.^23^

#### Breastfeeding acculturation and misconceptions about breastfeeding

Several of the narratives shed light on the reproduction or adoption of certain misconceptions regarding breastfeeding that have spread throughout Western culture, particularly since the second half of the 20^th^ century, based on the influence of so-called “bottle-feeding culture.” In other words, this could be thought of as a genuine breastfeeding acculturation,^61^ which has led to a worldwide pandemic of social hypogalactia,^23^ consistent with troublingly low rates of breastfeeding.^62^

We speak of misconceptions^63^ that have been socially and culturally transmitted (an erroneous and harmful breastfeeding socialization), along with highly ingrained opinions, as factors that contribute to the loss of breastfeeding culture and appropriate socialization into this biocultural practice. Moreover, these misconceptions have been revised and countered for some time from the perspective of feminist phenomenology.^2^^)^ They often come from healthcare professionals themselves (both inside and outside prison), from prison staff (educators, officials, etc.), and indeed from other inmates. This may lead to interference with initiating and continuing breastfeeding, with severe consequences for the women’s experiences. For example, frequent reference is made to a “*lack of milk*” or “*poor milk production*:”

*I said to the pediatrician* [at the hospital] *that he wasn’t getting full after I nursed and I wasn’t producing a lot of milk, and she told me to drink lots of liquids and to nurse him and give him the bottle. I was nursing and bottle feeding, but he would get so much gas, and when I told the pediatrician she said to me: “pick one or the other because you don’t produce a lot of milk… and if you’re giving him formula, give him a better formula.” And at a month and a half I was only giving him formula.* (Mother 3)

*When I nursed my baby she didn’t get full and it really hurt my breast* […] *They told me it was because I have low milk production.* (Mother 25)

We also encountered references to “incorrect” breast size or shape (even though there is scientific evidence^64^ that there is no physiological basis for this; all breasts are capable of milk production, except for a small percentage linked to certain conditions, with no statistical significance):

*I liked it. If I could have, I would have continued longer… but since I have this problem with one breast larger than the other I couldn’t continue on and I stopped.* (Mother 15)

Allusions to the supposed “*poor quality*” of breastmilk and to “*bad breasts”* could also be observed:

*I was only breastfeeding because I didn’t want to give him milk* [formula]*. When I had my one-month checkup they saw that he wasn’t gaining weight. So the pediatrician* [at the Mother Unit] *told me that I had to give him extra because it looked like my milk wasn’t filling him up.* (Mother 20)

*I was nursing for a month. I stopped because my breasts were no good. I didn’t have any milk. The baby was starving, he was crying and crying* […] *so I stuck him with the bottle.* (Mother 26)

We also registered references to prolonged breastfeeding,^65^ that is, the belief that past a certain age breastfeeding should be discontinued and is frowned upon:

*The educators told me that at his age it wasn’t a good idea to keep nursing him, because what we should be doing is strengthening him,* […] *like it doesn’t let him grow up. Like if you keep nursing he is still a baby, you don’t let him grow mentally.* (Mother 21)

Regarding the ingrained belief that breastfeeding is painful and that it is “*normal*” to feel pain, some of the mothers declared:

*I mean, we all get these cracks. Eventually they go away.* (Mother 12)

#### Situations related to obstetric violence

From several of the mothers’ narratives that were analyzed it was also possible to detect situations of obstetric violence exerted against them and/or their infants. As previously mentioned, this issue is structurally linked to the practice of breastfeeding and it has a direct and negative impact on its effective initiation.

Mothers reported having been subjected to obstetric violence throughout the entire perinatal period by healthcare personnel (both in prison facilities and in hospital settings), whether by action or omission, which led to serious consequences for their health:

*And I’ll never forget that I couldn’t have the baby until I got back the PCR test results. And I was having these contractions that were killing me. And all I remember is that they said “PCR negative,” because I was already dilated 7 cm. My water broke and the baby started to come. And I was just pushing him back in. Because they said that I had to wait. And I remember that the midwife said: “I’ll be right back, let me know if you need anything.” And when I heard that the PCR test came back negative, I pressed the red button and she said “alright, let’s get ready.” And I told her: “there’s no need to get ready.” I just opened my legs and she was just standing off to the side watching and I just pushed once and the baby came. I remember that he was slipping out of her hands. And she said to me: “how did you do that?” And I said: “I couldn’t hold it anymore.” I mean, she didn’t even have enough time to put gloves on.* (Mother 1)

The narratives also describe situations of mistreatment during the postpartum period, a particularly vulnerable stage for mothers who have just given birth. This is despite the growing body of scientific evidence showing that “the emotional state of the woman during pregnancy, childbirth, and postpartum has significant repercussions, both for her and for the baby she carries, for the physical and mental health of each and every member of the family, in the short and long term:”^66^

*The police… I’m in a bed, I just gave birth. And they are there on a sofa. With their cellphones and tablets at full volume, and you just gave birth. You’re alone, you don’t have anybody, and they are there shouting, laughing. It’s horrible. You go to the bathroom and if you take more than five minutes they start knocking on the door: “why are you taking so long?!” But, I mean… where am I going to go… through the toilet?* (Mother 1)

It is known that postpartum depression can manifest in 10% to 15% of women. Risk factors for postpartum depression, according to Antúnez et al., include:

…stress factors in daily life (conflicts with partners, having experienced stressful events during pregnancy, economic situation, relationship status…), lack of support, poor obstetric history [...], problems with breastfeeding, gestational age, hormonal changes, lack of sleep, and genetic predisposition.^67^

Other risk factors for postpartum depression include: birth by cesarean section, primiparity, and preexisting mental health disorders.^67^^)^ Studies have shown that women who face postpartum depression experienced stress factors in their lives during pregnancy and after childbirth.^67^^,^^68^^)^ Some of these circumstances were present in the narratives of incarcerated women, and many of them reported having experienced high stress levels. Similarly, several of the women reported having felt extreme sadness, and suspected or had a feeling that they could be experiencing postpartum depression, despite never having been diagnosed by a healthcare professional.

*The way I see it… I was going through depression.* (Mother 2)

They also reported being subjected to humiliating and degrading treatment, infringing on the most basic rights of mothers and infants, both through direct actions and through a failure to promote, encourage, and support breastfeeding, representing a specific form of bodily discrimination:

*Sometimes the police were there when I was nursing and they would say to me: “let’s go!” And I would say: “I’m not done yet.” And they’d reply: “we have to go.” And they* [the nurses] *would say to me: “don’t worry, we’ll fill up a bottle for him and feed him.” And I’d say: “ok, thank you.” I think above all it’s them* [the police]*, they are the ones who...* (Mother 1)

## CONCLUSIONS

The primary objective of this article, as mentioned above, was to analyze the breastfeeding experiences of incarcerated women in the Spanish penitentiary system in order to potentially detect perceptions of practices related to obstetric violence during the perinatal period. Furthermore, we sought to delve into the experience of breastfeeding during pregnancy, childbirth, and postpartum for both women and their infants incarcerated in different Mother Units across Spain, in order to document and more clearly understand a critical and problematic situation, and wherever possible formulate appropriate and informed recommendations.

Based on these objectives and the analysis of our main results, we find a need for public and penitentiary policies that align with WHO recommendations (infants should be exclusively breastfed for the first six months of life, followed by the introduction of nutritionally adequate and safe complementary foods, together with breastfeeding up to at least two years of age). Moreover, such policies should outline strategies to ensure breastfeeding, and also to put an end to the serious violations of human rights to which women are exposed - the right to food and physical, psychological, and emotional health, as well as sexual and reproductive rights, since lactation is an essential part of the sexual cycle in women who are mothers.

Therefore, specific policies should also be formulated and implemented to prevent violent practices (by action or omission) both in a physical sense (uninformed, inappropriate, non-consensual, non-consented acts) and a psychological sense (paternalistic, authoritarian, humiliating, degrading treatment), that are commensurate with obstetric violence and therefore constitute a violation of human rights.

It is necessary to provide up-to-date training and professional development on these issues for all parties involved during incarceration, so that they can ensure the well-being of mothers and infants during the perinatal period and have the tools to effectively and meaningfully implement actions to promote, protect, and support breastfeeding.

Similarly, there is an urgent need for perinatal mental health professionals in correctional facilities, who can respond to the specific needs of women who are serving sentences during this period. Although our research has identified difficulties for incarcerated mothers - in many ways analogous to those experienced by others - the crucial element revealed (emphasizing an intersectional perspective) is that incarceration adds to a complex web of discrimination, deepening and accentuating inequalities.

Although they are still partial and open-ended, the findings of this study make a valuable contribution to the body of knowledge on motherhood in prisons, on the (complex and delicate) perinatal period for mothers/infants in this situation, and ultimately on breastfeeding itself, a fundamental phenomenon for human health and a fundamental right both of all children and all women who become mothers. Therefore, it is necessary to continue advancing knowledge in this field to further explore this population and delve into the various issues related to our research objective that require further examination. For example, there should be a deeper exploration of experiences related to obstetric violence in general in correctional settings, and in particular the approach to perinatal mental health.

For these reasons, the main conclusion of this article has to do with the undeniable need to apply penitentiary policies with a gender-based, feminist perspective that protect the most basic rights of mothers and infants - in addition to attempting to eradicate the serious inequalities and discriminations suffered by incarcerated women - while offering an effective response to their situation in accordance with human rights, especially regarding their breastfeeding practice, which is also a basic human right.
